# Injectable Nano‐Micro Composites with Anti‐bacterial and Osteogenic Capabilities for Minimally Invasive Treatment of Osteomyelitis

**DOI:** 10.1002/advs.202306964

**Published:** 2024-01-17

**Authors:** Guanghua Lu, Gang Zhao, Shen Wang, Hanqing Li, Qiang Yu, Qi Sun, Bo Wang, Li Wei, Zi Fu, Zhenyu Zhao, Linshan Yang, Lianfu Deng, Xianyou Zheng, Ming Cai, Min Lu

**Affiliations:** ^1^ Department of Orthopaedics Shanghai Tenth People's Hospital Tongji University School of Medicine Shanghai 200072 P. R. China; ^2^ Department of Orthopaedics Shanghai Key Laboratory for Prevention and Treatment of Bone and Joint Diseases Shanghai Institute of Traumatology and Orthopedics Ruijin Hospital Shanghai Jiao Tong University School of Medicine Shanghai 200240 P. R. China; ^3^ Department of Plastic and Reconstructive Surgery Shanghai Ninth People's Hospital Shanghai Jiao Tong University School of Medicine Shanghai 200011 China; ^4^ Taikang Bybo Dental Shanghai 200001 P. R. China; ^5^ Department of Orthopedic Surgery Shanghai Sixth People's Hospital Affiliated to Shanghai Jiao Tong University School of Medicine Shanghai 200233 China

**Keywords:** bacterial capture, bacterial osteomyelitis, nano‐micro composite, oligonucleotide, osteogenesis

## Abstract

The effective management of osteomyelitis remains extremely challenging due to the difficulty associated with treating bone defects, the high probability of recurrence, the requirement of secondary surgery or multiple surgeries, and the difficulty in eradicating infections caused by methicillin‐resistant *Staphylococcus aureus* (MRSA). Hence, smart biodegradable biomaterials that provide effective and precise local anti‐infection effects and can promote the repair of bone defects are actively being developed. Here, a novel nano‐micro composite is fabricated by combining calcium phosphate (CaP) nanosheets with drug‐loaded GelMA microspheres via microfluidic technology. The microspheres are covalently linked with vancomycin (Van) through an oligonucleotide (oligo) linker using an EDC/NHS carboxyl activator. Accordingly, a smart nano‐micro composite called “CaP@MS‐Oligo‐Van” is synthesized. The porous CaP@MS‐Oligo‐Van composites can target and capture bacteria. They can also release Van in response to the presence of bacterial micrococcal nuclease and Ca^2+^, exerting additional antibacterial effects and inhibiting the inflammatory response. Finally, the released CaP nanosheets can promote bone tissue repair. Overall, the findings show that a rapid, targeted drug release system based on CaP@MS‐Oligo‐Van can effectively target bone tissue infections. Hence, this agent holds potential in the clinical treatment of osteomyelitis caused by MRSA.

## Graphical abstract

A smart nano‐micro “CaP@MS‐Oligo‐Van” composites with dual‐loaded Vancomycin (Van) and calcium phosphate (CaP) nanosheets using microfluidic techniques is developed. The injectability of the composites offers the possibility of minimally invasive treatment of methicillin‐resistant Staphylococcus aureus (MRSA)‐induced osteomyelitis. The multifunctional composites exhibit greater sensitivity and precision targeting to bacteria, exert additional antibacterial effects, inhibit the inflammatory response, and promote the bone tissue repair simultaneously.

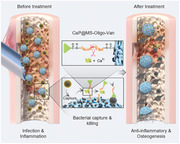

## 1 Introduction

Osteomyelitis is an inflammatory condition caused by pathogens and is accompanied by progressive bone destruction.^[^ ^1^ ^]^ Although a variety of microbes can cause osteomyelitis, ≈75% of cases are caused by opportunistic gram‐positive (G^+^) staphylococci.^[^ ^2^ ^]^ The treatment of osteomyelitis usually involves prolonged high‐dose antibiotic administration and extensive debridement surgery.^[^ ^3^, ^4^ ^]^ However, systemically injected antibiotics usually show poor bone penetration. This results in suboptimal local treatment, high blood levels of the antibiotic, and systemic toxicity. Some patients may even require extensive debridement surgeries, which can induce more trauma.^[^ ^5^, ^6^ ^]^ Thus, minimally invasive treatment has become a popular approach in the clinic, offering potential solutions to the limitations of traditional, ineffective osteomyelitis treatments.^[^ ^7^, ^8^ ^]^

Developments in microfluidic technology have enabled the synthesis of hydrogel microspheres with injectable properties and bolstered their application in bone tissue engineering.^[^ ^9^, ^10^ ^]^ Several materials have been used as drug carriers for the minimally invasive treatment of osteomyelitis. In particular, hydrogels have emerged as favorable candidates due to their biocompatibility, aqueous porous structure, similarity to organic matrices, and adaptable physicochemical properties.^[^ ^11^ ^]^ Using microfluidic technology, researchers can prepare porous hydrogel microspheres and precisely control their sizes, enabling the synthesis of injectable microspheres with an ideal size and uniform porous structure.^[^ ^12^, ^13^ ^]^ Hydrogel microspheres have been used for drug and cell delivery, leveraging their porous structure to provide a suitable surface area for cell adhesion and proliferation.^[^ ^13^, ^14^ ^]^ In addition, hydrogel microspheres have proven highly effective at trapping bacteria due to their rough fibrous surface and porous structure.^[^ ^15^ ^]^

Many studies indicate that the incidence of methicillin‐resistant *Staphylococcus aureus* (MRSA)‐induced osteomyelitis is increasing yearly.^[^ ^16^, ^17^ ^]^ Vancomycin (Van), a glycopeptide antibiotic with antibacterial activity against staphylococci, streptococci, and other G^+^ bacteria, is particularly effective against MRSA.^[^ ^18^ ^]^ Hence, it is the best bactericidal agent for the treatment of G^+^ bacterial infections. In addition, Van can achieve active bacterial capture by forming hydrogen bonds with the D‐alanine‐D‐alanine (D‐Ala‐D‐Ala) ligase expressed on the cell walls of G^+^ bacteria.^[^ ^19^, ^20^ ^]^ Although local drug delivery via targeted release is a safe approach for achieving effective local concentrations and reducing systemic drug exposure, the implementation of such delivery strategies remains challenging.^[^ ^21^, ^22^ ^]^ Interestingly, when antibiotics are covalently linked to biomaterial surfaces, and linkers or polymer chains (of appropriate flexibility/length) are present at the modification site, the antibiotics can exert bactericidal effects with minimal interference.^[^ ^23^, ^24^, ^25^ ^]^ Nevertheless, when such a surface modification approach is used, antibiotic action is often limited to the immediate surface of the implant. For example, Song et al.^[^ ^26^ ^]^ used Van covalently bound to polymers grafted on Ti6Al4V intramedullary needles to effectively inhibit the colonization/growth of *S. aureus* on the surface of metal implants. However, the covalently linked Van failed to eradicate bacterial growth in the tissue surrounding the site of osteomyelitis. This was because the covalently linked Van could not diffuse out from the surface of the biomaterial.^[^ ^27^ ^]^

Various advanced drug delivery systems currently leverage external stimuli such as pH, temperature, magnetic fields, and ultrasound to trigger drug release.^[^ ^28^, ^29^, ^30^, ^31^ ^]^ However, these systems are all inefficient at targeting infection sites to achieve precise antibiotic release.^[^ ^32^, ^33^, ^34^ ^]^ Interestingly, these drawbacks can be addressed by using a unique material sensitive to bacteria‐specific enzymes as a covalent linker. As we know, *S. aureus* can produce various enzymes including proteases, lipases, nucleases, collagenases, and hyaluronidases.^[^ ^35^ ^]^ Of these, micrococcal nuclease (MN) is the most common nuclease and serves as a biomarker to confirm the presence of *S. aureus* infection.^[^ ^36^ ^]^ MN can effectively and specifically degrade the single‐stranded forms of DNA and RNA in the presence of Ca^2+^.^[^ ^37^, ^38^, ^39^ ^]^ Thus, further modification using MN‐sensitive oligonucleotides (oligo) can address the disadvantages associated with the covalent surface modification of biomaterials. This can enable the precise release of antibiotics, reduce cytotoxicity due to the release of large doses of physically encapsulated antibiotics, and prevent bacterial resistance due to inadequate or delayed antibiotic release.

Because osteomyelitis typically causes severe bone destruction and bone loss, it is also essential to repair the bone tissue while treating the infection.^[^ ^4^, ^40^ ^]^ Various inorganic materials with biomedical properties, such as metal ion‐based biomaterials and bioglass,^[^ ^41^, ^42^ ^]^ have been widely used in bone tissue repair engineering.^[^ ^41^ ^]^ Osteocytes have a higher affinity for calcium phosphate (CaP) than for metals or polymers.^[^ ^43^ ^]^ Bones are mainly composed of hierarchically assembled nanohydroxyapatite (30–50 nm long, 20–25 nm wide, and 1.5–4.0 nm thick) and an organic matrix.^[^ ^44^ ^]^ Recent studies have shown that the morphology and structure of nano‐hydroxyapatite can directly influence the osteogenic microenvironment, thus regulating bone regeneration.^[^ ^20^, ^45^ ^]^ It is clear that nano‐hydroxyapatite and organic matrices have crucial functions in the regeneration of bone tissues. Therefore, using biomimetic hydrogels and CaP to simulate inorganic and organic matrices simultaneously could effectively induce cell attachment to bone tissue, thereby supporting cell differentiation and proliferation.^[^ ^46^, ^47^ ^]^

**Scheme 1:**
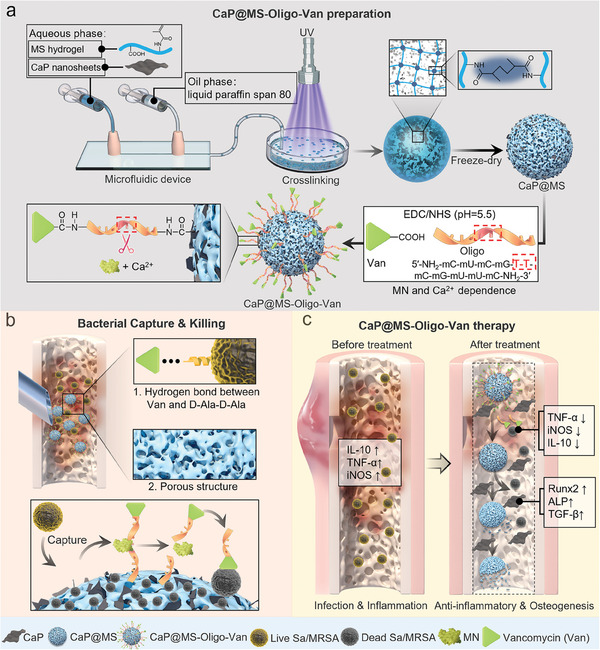
Schematic illustration of CaP@MS‐Oligo‐Van synthesis and its therapeutic effects in osteomyelitis. a) Design and preparation of injectable and smart nano‐micro CaP@MS‐Oligo‐Van composites. b) Schematic showing bacterial capture and killing. The bacteria could be adsorbed and captured by the CaP@MS‐Oligo‐Van composites due to their abundant porous structures and the hydrogen bonds between Van and the bacterial cell wall. The Van was released from the oligo of CaP@MS‐Oligo‐Van due to the shearing action of MN secreted by pathogenic *S. aureus* or MRSA in the presence of Ca^2+^. This Van further killed the bacteria. c) The application of nano‐micro CaP@MS‐Oligo‐Van composites for the treatment of osteomyelitis. The composites effectively inhibited the expression of inflammatory cytokines (TNF‐α, iNOS, and IL‐10) and promoted osteogenesis (RUNX2, ALP, and TGF‐β).

Herein, we prepared injectable smart nano‐micro CaP@MS‐Oligo‐Van composites with dual‐loaded Van and CaP nanosheets using microfluidic techniques. We covalently functionalized GelMA hydrogel microspheres with Van via MN‐sensitive sequence‐specific oligo linkers (5′‐NH_2_‐mC‐mU‐mC‐mG‐T‐T‐mC‐mG‐mU‐mU‐mC‐NH_2_‐3′) (**Scheme** **1**a). In the presence of *S. aureus* or MRSA, the smart CaP@MS‐Oligo‐Van composites could achieve active bacterial capture due to their porous structures and the formation of hydrogen bonds between Van and the bacteria. Meanwhile, the MN secreted by *S. aureus*/MRSA could cleave the oligo linker in the presence of Ca^2+^ to achieve the precise release of Van (Scheme 1b). Accordingly, bactericidal effects could be achieved at the site of infection, and associated inflammatory factor expression (TNF‐α, iNOS, and IL‐10) could be reduced. In addition, the CaP nanosheets—released slowly through the pores of the composites—could regulate the osteogenic microenvironment, upregulating relevant osteogenic genes and growth factors (RUNX2, ALP, COL‐1, OCN, and TGF‐β) to promote osteogenesis (Scheme 1c). Overall, this study describes the development of a new antibacterial carrier consisting of hydrogel microspheres covalently linked to antibiotics that simultaneously induces precise antibacterial and osteogenic actions in vivo for the treatment of osteomyelitis.

## 2 Results and Discussion

### 2.1 Characterization of CaP Nanosheets and Nano‐Micro CaP@MS‐Oligo‐Van Composites

GelMA was used to prepare size‐controlled hydrogel microspheres (MS) via microfluidic technology. CaP nanosheets were physically encapsulated in the aqueous phase during the preparation of MS to obtain CaP@MS. Then, an oligo linker (5′‐NH_2_‐mC‐mU‐mC‐mG‐T‐T‐mC‐mG‐mU‐mU‐mC‐NH_2_‐3′) was employed to covalently connect Van with CaP@MS using EDC/NHS chemistry. Subsequently, nano‐micro CaP@MS‐Oligo‐Van composites were obtained (Figure S1, Supporting Information). The composite microspheres had a uniform size, stable morphology, and excellent mono‐dispersibility, indicating their superior injectability.^[^ ^48^, ^49^ ^]^ In addition, the injectability of the CaP@MS‐Oligo‐Van was verified through extrusion experiments (Video S1, Supporting Information).

**Figure 1:**
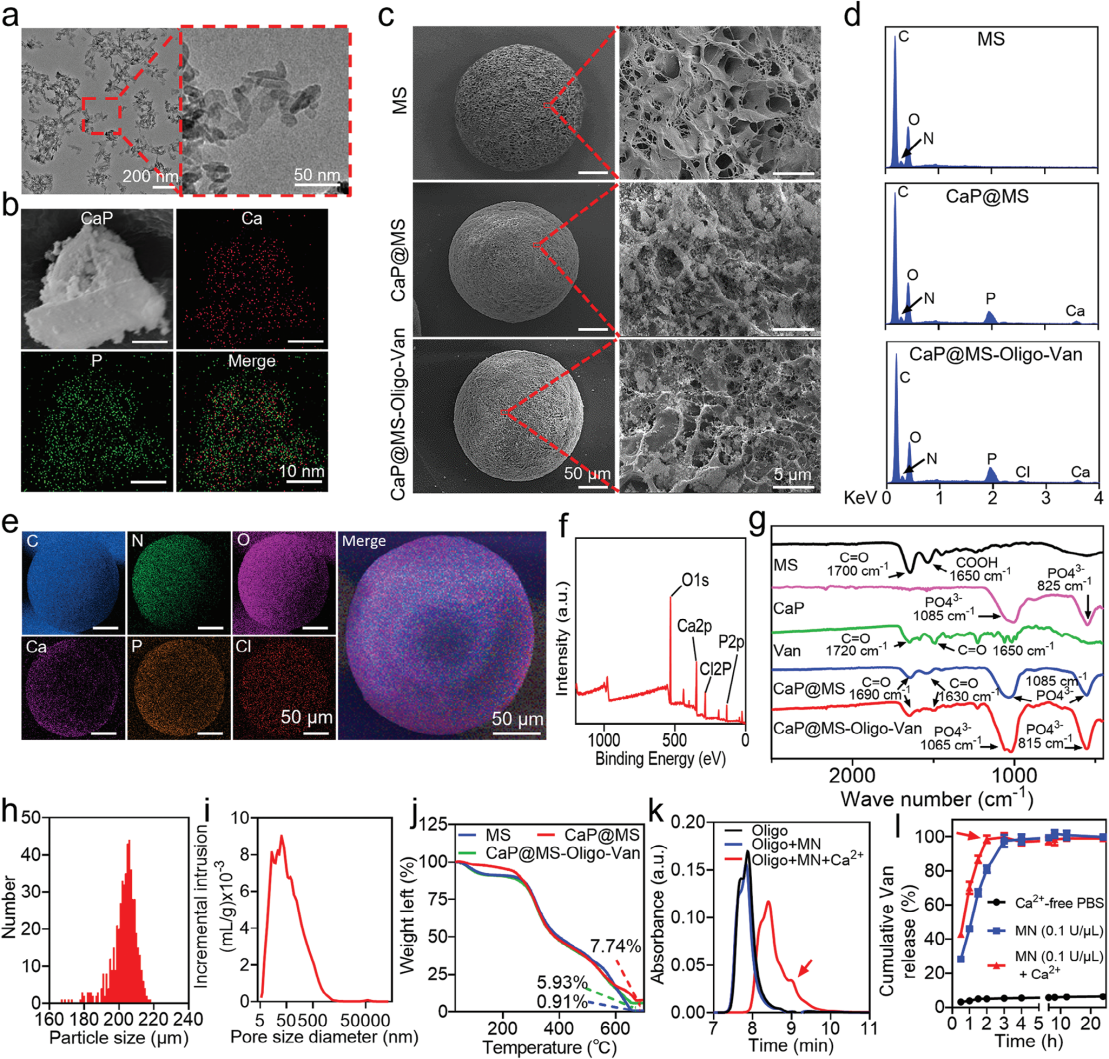
Characteristics of CaP nanosheets and nano‐micro CaP@MS‐Oligo‐Van composites. a,b) Representative TEM images (a) and EDS elemental mapping images (b) of CaP nanosheets. c,d) Representative SEM images (c) and energy spectrum (d) of MS, CaP@MS, and CaP@MS‐Oligo‐Van composites, confirming the presence of Ca, P, and Cl in CaP@MS‐Oligo‐Van. e,f) Representative EDS elemental mapping images (e) and XPS spectrum (f) of the prepared CaP@MS‐Oligo‐Van composites, further verifying the presence of C, N, O, Ca, P, and Cl in the composites. g) FT‐IR spectra of Van, CaP nanosheets, MS, CaP@MS, and CaP@MS‐Oligo‐Van. h,i) Distribution of the particle size (h) and pore diameter (i) of the CaP@MS‐Oligo‐Van composites. j) TGA curves of MS, CaP@MS, and CaP@MS‐Oligo‐Van composites. k) GPC trace validating the site‐specific cleavage of the oligo by MN in the presence of Ca^2+^. l) Ca^2+^ accelerated the MN‐responsive release of Van from the oligo of CaP@MS‐Oligo‐Van. Images are representative of at least three independent experiments. The results in Figure l are presented as the mean ± SD of five independent experiments.

Transmission electron microscopy (TEM, Figure 1a) and scanning electron microscopy (SEM, **Figure** **1**b) revealed that the synthesized CaP nanomaterials had sheet‐like structures with similar lengths ranging from 30 nm to 50 nm. Elemental mapping demonstrated the presence of the elements Ca and P in the CaP nanosheets (Figure 1b), validating the successful synthesis of CaP nanosheets. In addition, SEM images revealed that numerous porous and fibrous structures were present in the prepared hydrogel microspheres. Moreover, dark‐colored CaP nanosheets were only visible on the surface of CaP@MS and CaP@MS‐Oligo‐Van samples (Figure 1c). The porous and fibrous structures on the surface of the hydrogel microspheres not only enhanced the area of contact with microbes or cells,^[^ ^50^ ^]^ but also contributed to bacterial capture and cell adhesion capabilities.^[^ ^51^ ^]^ Furthermore, the macroscopic appearance of the composite microspheres was nearly identical before (Figure 1c) and after (Figure S1, Supporting Information) rehydration. The incorporation of CaP nanosheets and Van did not compromise the structure and morphology of the hydrogel microspheres, maintaining their excellent injectability.^[^ ^49^ ^]^ Energy dispersive X‐ray spectroscopy (EDS) analysis further demonstrated the presence of the corresponding Ca, P, and Cl elements in the CaP@MS‐Oligo‐Van samples (Figure 1d). Herein, Ca and P were specific to CaP nanosheets and Cl was specific to Van. Additionally, the elemental mapping analysis of MS, CaP@MS, and CaP@MS‐Oligo‐Van was also conducted. Only three elements, i.e., C, N, and O, were observed in MS (Figure S2a, Supporting Information). The content of Ca and P was discovered in the CaP@MS samples due to the presence of encapsulated CaP nanosheets (Figure S2b, Supporting Information). Furthermore, the elements C, N, O, Ca, P, and Cl were uniformly distributed in the CaP@MS‐Oligo‐Van samples (Figure 1e). Finally, X‐ray photoelectron spectroscopy (XPS) verified that the aforementioned elements were present in the synthesized composites. This confirmed the successful synthesis of CaP@MS‐Oligo‐Van (Figure 1f; Figure S3a–f, Supporting Information).

Fourier transform infrared (FT‐IR) spectroscopy was subsequently conducted to understand the chemical structures of the composites. As shown in Figure 1g, in MS, a vibrational absorption peak corresponding to C═O appeared at 1700 cm^−1^, and a telescopic vibrational absorption peak corresponding to the C═O of COOH appeared at 1650 cm^−1^. CaP nanosheets showed the telescopic vibrational absorption peak of PO_4_^3−^ at 1085 cm^−1^ and the bending vibrational absorption peak of the fingerprint region at 825 cm^−1^. Van had the vibrational absorption peak of C═O at 1720 cm^−1^ and the telescopic vibrational absorption peak of C═O at 1650 cm^−1^. When CaP nanosheets and MS were compounded to generate CaP@MS, the vibrational absorption peak of C═O appeared at 1690 cm^−1^, the telescopic vibrational absorption peak of C═O appeared at 1630 cm^−1^, and the telescopic vibrational absorption peak of PO_4_^3−^ appeared at 1085 cm^−1^. These findings also indicated the successful compounding of CaP@MS. CaP@MS‐Oligo‐Van showed the vibration absorption peak of PO_4_^3−^ at 1065 cm^−1^, a bending vibration absorption peak at 815 cm^−1^ in the fingerprint region, the vibration absorption peak of C═O at 1695 cm^−1^, and the vibration absorption peak of C═O at 1633 cm^−1^. This further confirmed the successful preparation of the nano‐micro composites (Figure 1g).

The microfluidic emulsion approach enables the fabrication of extremely monodisperse microspheres of variable sizes.^[^ ^52^ ^]^ The obtained nano‐micro CaP@MS‐Oligo‐Van composites had an average diameter of 203 µm (170–240 µm) (Figure 1h), consistent with the results of SEM (Figure 1c) and elemental mapping (Figure 1e). In addition, the surface of the composites had extensive pores with diameters ranging from 5 to 5000 nm and a porosity of up to 81.68% (Figure 1i). This was also consistent with the SEM images (Figure 1c). Of note, GelMA possesses excellent self‐degradation performance.^[^ ^53^ ^]^ Accordingly, the prepared CaP@MS‐Oligo‐Van composites showed almost complete degradation after 6 weeks in MEM‐*α* culture medium (Figure S4, Supporting Information).

Thermogravimetric curves were recorded using a thermal gravimetric analyzer (TGA). These curves were used to estimate the loading rates of CaP nanosheets in CaP@MS and CaP@MS‐Oligo‐Van samples (Figure 1j). The weight loss ratio in each group was calculated after heating from room temperature to 700 °C. Thermogravimetric curves indicated that the loading rates of CaP nanosheets in CaP@MS and CaP@MS‐Oligo‐Van were 6.83% and 5.02%, respectively. The lower loading rate of CaP nanosheets in CaP@MS‐Oligo‐Van could be due to partial loss during the grafting of Van. By detecting the total amount of unreacted Van in the supernatant and dialysate, based on the calibration curve (Figure S5, Supporting Information), we calculated that the drug loading rate of Van in CaP@MS‐Oligo‐Van was ≈95.33 ± 5.03 µg mg^−1^ (Table S1, Supporting Information).

We further confirmed the site‐specific cleavage of the oligo in CaP@MS‐Oligo‐Van by MN under different Ca^2+^ conditions. As shown in Figure 1k, when the composites were placed in PBS containing MN, the oligos were cleaved into two fragments if Ca^2+^ was present (arrow). However, there was essentially no cleavage in the absence of Ca^2+^. In the drug‐release test (Figure 1l), the release of Van from CaP@MS‐Oligo‐Van was negligible when MN was not added. This showed that the oligo linker was adequately stable (black line). Due to the micro‐soluble nature of CaP in aqueous solution,^[^ ^54^ ^]^ CaP@MS‐Oligo‐Van could spontaneously release Ca^2+^ and activate the enzyme activity of MN. This resulted in the rapid release of Van even in Ca^2+^‐free PBS (blue line). Notably, the responsive release of Van in PBS was further accelerated by the addition of a physiological concentration of Ca^2+^, allowing almost complete release of Van within 2 h (red line). As shown in Figure S6a (Supporting Information), responsive release of Van accelerated with increasing concentrations of MN, indicating that the more severe the localized bone tissue was infected, the stronger the responsive release of Van was. Additionally, we also inactivated MN by heating it to 100 °C for 5 min before performing the responsive release experiment. As shown in Figure S6b (Supporting Information), inactivated MN was incapable of promoting the release of Van. This confirmed that the enzymatic cleavage of oligo by MN is a critical factor for the targeted release of Van. Together, the findings showed that CaP@MS‐Oligo‐Van can rapidly and precisely release Van without relying on exogenous Ca^2+^, addressing the inability to release Van in Ca^2+^‐free environments.

### 2.2 Biocompatibility of Nano‐Micro CaP@MS‐Oligo‐Van Composites

**Figure 2:**
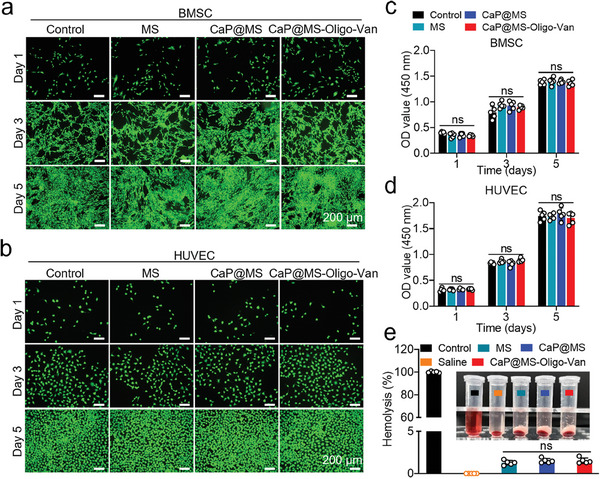
Biocompatibility of the nano‐micro CaP@MS‐Oligo‐Van composites. a,b) Representative live/dead fluorescence images of BMSCs (a) and HUVECs (b). The BMSCs and HUVECs were cocultured with MS, CaP@MS, and CaP@MS‐Oligo‐Van for 1, 3, and 5 days and further processed using live/dead staining. The viable and dead cells were visualized using calcein‐AM (green) and PI (red) staining, respectively. c,d) The cell viabilities of BMSCs and HUVECs treated with the CaP@MS‐Oligo‐Van composites were further evaluated using the CCK‐8 assay. (e) In vitro hemolysis test of the CaP@MS‐Oligo‐Van composites. Images are representative of at least three independent experiments. The results in Figure 2 are presented as the mean ± SD of five independent experiments. ns, no significance.

Bone mesenchymal stem cells (BMSCs) and human umbilical vein endothelial cells (HUVECs) were respectively co‐cultured with MS, CaP@MS, and CaP@MS‐Oligo‐Van for 1, 3, and 5 days in 24‐well plates. Live/dead staining at these time points showed that all co‐cultured BMSCs and HUVECs were in good condition. Moreover, there was no obvious difference in cell survival between these groups (**Figure** **2**a,b; Figures S7 and S8, Supporting Information). Further, the CCK‐8 assay confirmed that the composites had no significant effects on cell proliferation (Figure 2c,d), demonstrating their good biocompatibility. Finally, hemolysis assays were also performed, with deionized water serving as a positive control and saline serving as a negative control.^[^ ^55^ ^]^ After the co‐incubation of red blood cells (RBCs) with MS, CaP@MS, and CaP@MS‐Oligo‐Van, no significant hemolysis was detected. Moreover, the hemolysis rate was significantly lower than 5% under all conditions. This verified that the composites had good hemocompatibility (Figure 2e).

### 2.3 In Vitro Antibacterial Performance of Nano‐Micro CaP@MS‐Oligo‐Van Composites

**Figure 3:**
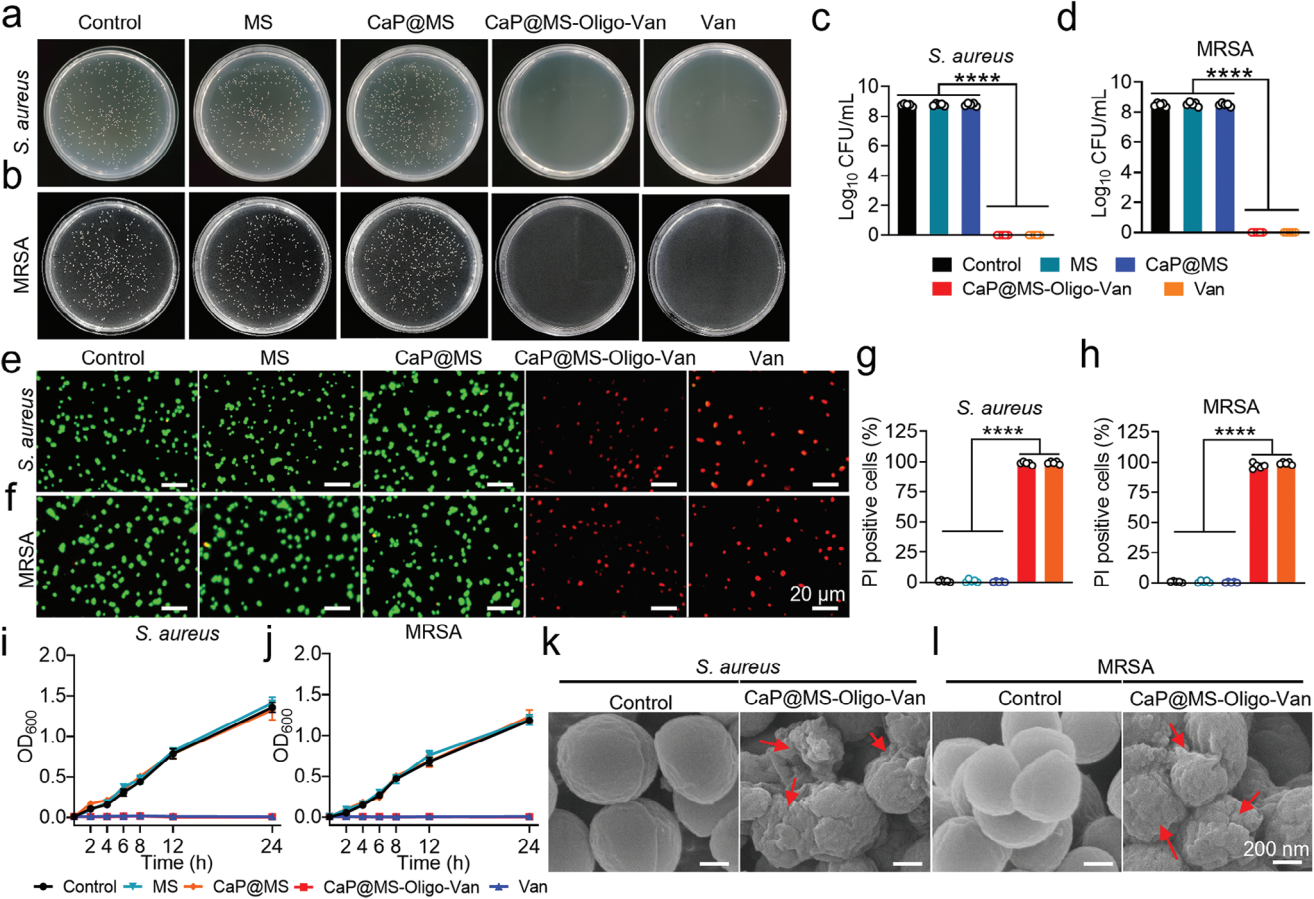
In vitro antibacterial activity of the nano‐micro CaP@MS‐Oligo‐Van composites. a,b) Representative colony plate images of *S. aureus* (a) and MRSA (b) after culture on BHI medium alone (control) and BHI medium containing MS, CaP@MS, CaP@MS‐Oligo‐Van, and Van alone. c,d) CFU counts for *S. aureus* (c) and MRSA (d) after the abovementioned treatments. e,f) Representative live/dead fluorescence images of *S. aureus* (e) and MRSA (f) after the abovementioned treatments. The viable and dead bacteria were stained with SYTO 9 (green) and PI (red), respectively. g,h) Percentage of PI‐positive *S. aureus* (g) and MRSA cells (h). The counts in Figure 3g,h were obtained based on the images shown in Figure 3e,f, respectively. i,j) The 24‐h growth curves of *S. aureus* (i) and MRSA (j) in the presence of MS, CaP@MS, and CaP@MS‐Oligo‐Van composites. k,l) Representative SEM images of *S. aureus* (K) and MRSA (l) before and after CaP@MS‐Oligo‐Van composite treatment. Red arrows show cell collapse and cell wall and membrane damage. Images are representative of at least three independent experiments. The results in Figure 3c,d,g–j are presented as the mean ± SD of five independent experiments. ^****^ *p* < 0.0001.

The antibacterial activities of MS, CaP@MS, and CaP@MS‐Oligo‐Van against *S. aureus* and MRSA were studied. Free Van was utilized as a positive control to determine whether the Van grafted on CaP@MS‐Oligo‐Van lost some of its antibacterial capabilities. *S. aureus* and MRSA inoculates were cultured in BHI medium containing CaP@MS‐Oligo‐Van and free Van. After 6 h of co‐incubation, the medium in both the CaP@MS‐Oligo‐Van and Van groups remained clear, appearing similar to sterile BHI medium (Figure S9a,b, Supporting Information). However, the other groups showed apparent turbidity due to the growth of *S. aureus* and MRSA. The outcomes of the OD_600_ assay for each supernatant are displayed in Figure S9c,d (Supporting Information). Standard plate counts demonstrated the excellent bactericidal abilities of CaP@MS‐Oligo‐Van and free Van against both *S. aureus* and MRSA. Specifically, no colonies were identified in the BHI plates in these groups (**Figure** **3**a‐d).

In addition, after 2 h of co‐incubation in PBS, live/dead staining was performed (Figure 3e,f; Figures S10 and S11, Supporting Information). The control, MS, and CaP@MS groups almost exclusively exhibited green fluorescence. This indicated the low bactericidal activities of these agents against *S. aureus* and MRSA. The CaP@MS‐Oligo‐Van and Van groups, in contrast, almost exclusively showed red fluorescence, demonstrating that a majority of the bacteria in these groups were dead. The percentage of PI‐positive dead bacteria in each group was also calculated (Figure 3g,h). Notably, over 95% of *S. aureus* and MRSA cells were dead after treatment with CaP@MS‐Oligo‐Van and Van.

The effects of the nano‐micro CaP@MS‐Oligo‐Van composites on the growth curves of *S. aureus* and MRSA in BHI medium were further investigated (Figure 3i,j). During the 24 h co‐culture period, the MS and CaP@MS groups showed a considerable increase in OD_600_ values, with no significant differences when compared with the control group. This indicated that both MS and CaP@MS treatments could not inhibit bacterial growth. In contrast, CaP@MS‐Oligo‐Van and Van completely inactivated *S. aureus* and MRSA in BHI medium, showing nearly uniform OD_600_ values during the 24 h of co‐culture (Figure 3i,j).

SEM was employed to observe the morphology changes in *S. aureus* and MRSA after CaP@MS‐Oligo‐Van treatment. As shown in Figure 3k,l, *S. aureus*/MRSA in the control group exhibited round or oval structures and had an intact smooth surface. Meanwhile, bacteria exposed to CaP@MS‐Oligo‐Van underwent severe morphological alterations, including cell membrane rupture, crumpling, and loss of cellular integrity. Hence, these bacteria had lost their vitality.

In summary, all the above results (displayed in Figure 3) revealed that CaP@MS‐Oligo‐Van exerts strong bactericidal effects against *S. aureus* and MRSA. Moreover, grafted Van retains its antibacterial activity after chemical modification. The sub‐MIC antibiotic level is one of the key factors in the development of bacterial resistance.^[^ ^56^ ^]^ However, CaP@MS‐Oligo‐Van could release Van far above the MIC (2 µg mL^−1^). Hence, the composites could not only rapidly inactivate bacteria, but also reduce the emergence of bacterial resistance.

### 2.4 Ability of Nano‐Micro CaP@MS‐Oligo‐Van Composites to Capture *S. aureus* and MRSA

Hydrogels are capable of efficiently capturing bacteria owing to the network of rough fibers and porous surface structure they possess.^[^ ^15^ ^]^ The rough fiber network and abundant porous structures on the surface of the nano‐micro CaP@MS‐Oligo‐Van composites were observed using SEM in this study (Figure 1c). Furthermore, studies show that Van can form hydrogen bonds with D‐Ala‐D‐Ala on the cell wall of G^+^ *S. aureus* and MRSA.^[^ ^19^, ^57^ ^]^ This enhances the ability of hydrogel microspheres to capture bacteria. Hence, the ability of nano‐micro CaP@MS‐Oligo‐Van composites to capture *S. aureus* and MRSA was examined in this study.

**Figure 4:**
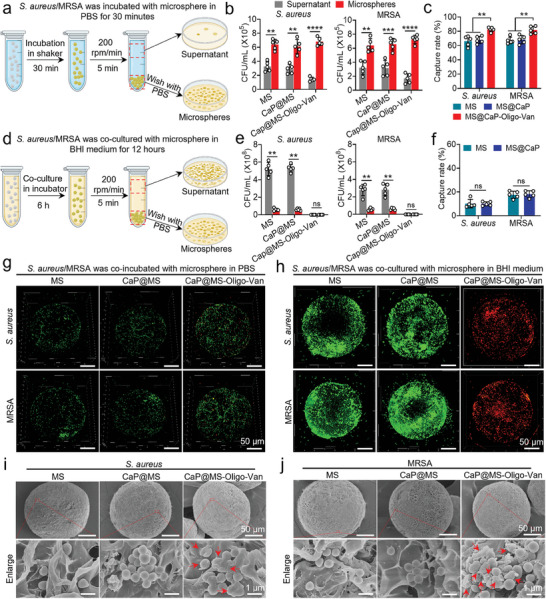
Ability of nano‐micro CaP@MS‐Oligo‐Van composites to capture *S. aureus* and MRSA. Schematics of the experimental designs used to test bacterial capture in PBS a) and BHI medium d). (a and ,d) Created with BioRender.com. Briefly, *S. aureus*/MRSA suspensions (10^6^ CFU mL^−1^) were co‐incubated with MS, CaP@MS, or CaP@MS‐Oligo‐Van in PBS for 30 min (a) or in BHI medium for 6 h (d). After treatment, the MS/composites and supernatants were separated via centrifugation, and the bacterial colonies in the two portions were quantified using standard plate counts. b,e) CFU counts of *S. aureus* (left) and MRSA (right) from the composites and supernatants of PBS (b) and BHI medium (e). c,f) Capture rates of the CaP@MS‐Oligo‐Van composites for *S. aureus* and MRSA in PBS (c) and BHI medium (f). The data in Figure 4c,f were calculated according to the results shown in Figure 4b,e, respectively. g,h) Representative live/dead fluorescence images of *S. aureus* (upper) and MRSA (down) adsorbed on the surfaces of the composites in PBS (g) and BHI medium (h). The *S. aureus*/MRSA suspension was co‐incubated with MS, CaP@MS, and CaP@MS‐Oligo‐Van in PBS for 30 min (g) and in BHI medium for 6 h (h). The composites were collected by centrifugation and stained with SYTO 9/PI, and then further observed using CLSM. The viable and dead bacteria were stained with SYTO 9 (green) and PI (red), respectively. i,j) Representative SEM images showed that the majority of *S. aureus* and MRSA cells were captured and were present on the surfaces of the composites. Arrows indicate the changes in cell morphology and cell wall and membrane damage. Images are representative of at least three independent experiments. The results in Figure 4b,c,e,f are presented as the mean ± SD of five independent experiments. ^**^ *p* < 0.01, ^***^ *p* < 0.001, ^****^ *p* < 0.0001.

We co‐incubated MS, CaP@MS, and CaP@MS‐Oligo‐Van with *S. aureus* and MRSA (1 × 10^6^ CFU mL^−1^) for 0.5 h in PBS to determine their bacterial capture capacity. We utilized the standard plate count method to count the bacteria in the supernatant and the bacteria that had been captured on the microspheres (**Figure** **4**a). As shown in Figure 4b and Figure S12a,b (Supporting Information), there were significantly more bacteria captured by the microspheres than there were free bacteria in the supernatant. Statistical analysis (Figure 4c) demonstrated that the capture rates for *S. aureus* in the MS and CaP@MS groups were 66.27 ± 7.132% and 67.90 ± 5.044%, respectively. Moreover, the capture rates for MRSA in these groups were 67.08 ± 4.696% and 68.24 ± 5.810%, respectively. In comparison, the capture rates for *S. aureus* and MRSA were significantly higher in the CaP@MS‐Oligo‐Van group (82.59 ± 3.182% and 81.73 ± 5.281%, respectively). Following co‐incubation, the composites were examined using live/dead staining and observed with confocal laser scanning microscopy (CLSM). The results are displayed in Figure 4g and Figures S13a and S14a (Supporting Information). Notably, captured *S. aureus* and MRSA could be seen on the surface of the microspheres. Fluorescence quantification results showed that the area of fluorescence was significantly higher in the CaP@MS‐Oligo‐Van group than in the MS and CaP@MS groups (Figures S13b and S14b, Supporting Information). The rich micrometer‐scale pore structure of the hydrogel microspheres played a crucial role in capturing bacteria. Further, Van enhanced the bacterial capture ability of CaP@MS‐Oligo‐Van. Overall, the effectiveness of this composite in capturing bacteria can be attributed to physical trapping and chemical bonds, which are significantly stronger than electrostatic interactions.^[^ ^22^ ^]^

We further extended the co‐incubation period of bacteria and composites to 12 h in BHI medium and allowed the bacteria to freely propagate to the stable phase to detect the upper limit of bacterial capture (Figure 4d). The CFU counts of free bacteria in the supernatant and bacteria captured on the microspheres are shown in Figure 4e and Figure S15a,b (Supporting Information). The number of bacteria captured by the composites increased significantly with the increase in the bacterial concentration. The counts of *S. aureus* captured in the MS and CaP@MS groups were 5.80 ± 1.50 × 10^7^ CFU mL^−1^ and 5.96 ± 1.18 × 10^7^ CFU mL^−1^, respectively. Moreover, the respective counts of captured MRSA were 5.85 ± 1.40 × 10^7^ CFU mL^−1^ and 6.10 ± 1.21 × 10^7^ CFU mL^−1^. Notably, the number of colonies could not be evaluated in the CaP@MS‐Oligo‐Van group. This was because the powerful antibacterial effects of this composite led to the death of almost all bacteria. Overall, the results showed that as the concentration of bacteria increased, the microspheres could capture a greater number of bacteria (higher than 1 × 10^6^ CFU mL^−1^) until their pores were filled with bacteria. Figure 4f displays the data on bacterial capture rate. In the MS and CaP@MS groups, the capture rates for *S. aureus* were 10.14 ± 3.54% and 10.02 ± 1.68%, respectively, and those for MRSA were 17.58 ± 3.01% and 18.08 ± 2.84%, respectively. MRSA was captured at a higher rate than *S. aureus* because its bacterial concentration in the supernatant during growth to the stable phase was lower than that of *S. aureus*. However, there was no significant difference in the amount of *S. aureus* and MRSA captured by the composites.

The composites were further examined using live/dead staining after co‐incubation and observed using CLSM. The results are shown in Figure 4h and Figures S16a and S17a (Supporting Information). Massive amounts of captured *S. aureus* and MRSA could be observed in each group of composites. The MS and CaP@MS groups exhibited green fluorescence nearly exclusively. This demonstrated that the microspheres had successfully trapped a significant amount of bacteria but were unable to eliminate them. In contrast, the CaP@MS‐Oligo‐Van group exhibited red fluorescence nearly exclusively, demonstrating that CaP@MS‐Oligo‐Van successfully trapped and eliminated a significant amount of bacteria. Quantification analysis showed that the CaP@MS‐Oligo‐Van group exhibited significantly higher levels of red fluorescence than the MS and CaP@MS groups. Meanwhile, the green fluorescence in the CaP@MS‐Oligo‐Van group was weak due to the inhibition of bacterial proliferation by the released Van (Figures S16b,c and S17b,c, Supporting Information).

The morphologies of the *S. aureus* and MRSA captured by the composites were observed using SEM. As shown in Figure 4i,j, in the MS and CaP@MS groups, the bacteria on the surface of the microspheres showed an intact morphology. However, the bacteria on the surface of CaP@MS‐Oligo‐Van exhibited severe deformation and rupture, indicating that the captured bacteria had been destroyed.

### 2.5 In Vitro Osteogenic Potential of Nano‐Micro CaP@MS‐Oligo‐Van Composites

**Figure 5:**
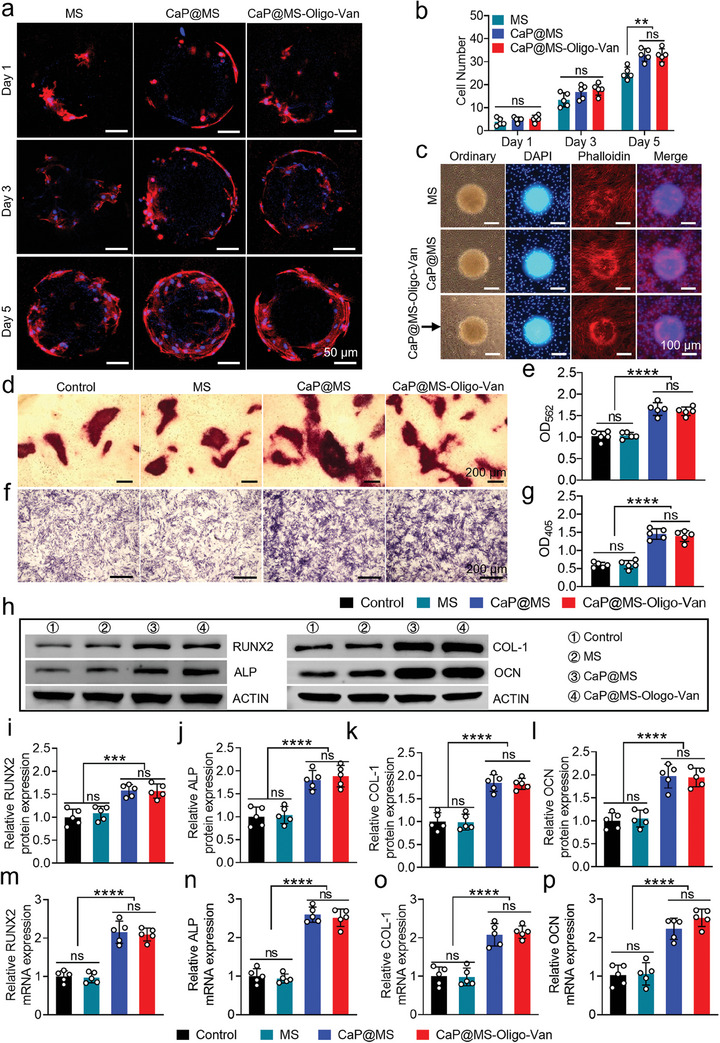
Biocompatibility of the nano‐micro CaP@MS‐Oligo‐Van composites with BMSCs, and their osteogenic potential. a,b,c) The effects of the CaP@MS‐Oligo‐Van on the proliferation behaviors of BMSCs on day 1, 3, and 5. The BMSCs on the surface of MS, CaP@MS, and CaP@MS‐Oligo‐Van were stained with Phalloidin/DAPI and observed using CLSM. Blue staining (DAPI) indicates the nuclei, and red staining (Phalloidin) indicates the cytoskeleton. Representative Phalloidin/DAPI fluorescence images of BMSCs on composites (a) and in 96‐well plates (c) after 1, 3, and 5 days of co‐incubation are presented. The numbers of BMSCs on the composites on day 1, 3, and 5 were statistically analyzed (b). d,e) Representative Alizarin Red S staining images (d) of BMSCs, and quantitative evaluation of mineralized nodules (e) after 14 days of co‐incubation between BMSCs and the composites. f,g) Representative ALP staining images (f) of BMSCs, and quantitative evaluation of ALP activity (g) after 7 days of co‐incubation between BMSCs and the composites. h,i,j) Protein expression of RUNX2, ALP, COL‐1, and OCN in BMSCs after co‐incubation with composites (h); the semi‐quantitative analysis of RUNX2 (i), ALP (j), COL‐1 k), and OCN protein l) expression is presented. m–p) qRT‐PCR analysis of the gene expression of RUNX2 (m), ALP (n), COL‐1 (o), and OCN (p) in BMSCs after co‐incubation with composites. Images are representative of five independent experiments. The results in Figure 5b,e,g,i–p are presented as the mean ± SD of five independent experiments. ^**^ *p* < 0.01, ^***^ *p* < 0.001, ^****^ *p* < 0.0001. ns, no significance.

We co‐incubated MS, CaP@MS, and CaP@MS‐Oligo‐Van with BMSCs for 1, 3, and 5 days. Then, we employed phalloidin and DAPI staining to observe the migration behavior of BMSCs. As shown in **Figure** **5**a–c and (Supporting Information Figure S18a–c), quantitative evaluation showed that the number of BMSCs on the composites gradually increased with the extension of the co‐incubation duration, demonstrating the excellent biocompatibility of the composites. Additionally, after 5 days of co‐incubation, there were significantly more BMSCs on microspheres in the CaP@MS and CaP@MS‐Oligo‐Van groups than in the MS group. Several studies have revealed that cells have a higher affinity for CaP (both in vitro and in vivo) than for other substrates, such as metals or polymers. Ca^2+^ release from CaP nanosheets is also known to stimulate osteoblast attachment and further promote cell adhesion, migration, proliferation, and differentiation.^[^ ^43^ ^]^

The ability of the nano‐micro CaP@MS‐Oligo‐Van composites to stimulate the mineralization of BMSCs was further analyzed using Alizarin Red S staining.^[^ ^58^ ^]^ CaP@MS and CaP@MS‐Oligo‐Van generated a substantial number of mineralized nodules (Figure 5d). Quantitative analysis confirmed that the composites containing CaP nanosheets could improve osteogenic activity in BMSCs (Figure 5e). We further utilized alkaline phosphatase (ALP) staining to detect ALP expression.^[^ ^59^, ^60^ ^]^ The results of ALP staining after 7 days of induction culture are shown in Figure 5f. The CaP@MS and CaP@MS‐Oligo‐Van groups exhibited more blue nodules and a darker color. Quantitative evaluation (Figure 5g) also demonstrated that CaP@MS and CaP@MS‐Oligo‐Van significantly promoted the expression of ALP.

Additionally, we investigated the effect of nano‐micro composites on osteogenesis‐related proteins and genes.^[^ ^61^, ^62^ ^]^ The expression of ALP, RUNX2, COL‐1, and OCN at the protein (Figure 5h–l) and gene level (Figure 5m–p) was identified on day 14 following the co‐incubation of composites and BMSCs. According to qRT‐PCR and western blot analysis, the expression of ALP, RUNX2, COL‐1, and OCN was considerably higher in the CaP@MS and CaP@MS‐Oligo‐Van groups than in the MS and control groups. Studies have shown that the shape and structure of artificial CaP nanosheets can directly impact the osteogenic microenvironment, further affecting the process of bone regeneration, including osteoblast attachment and migration and osseointegration.^[^ ^20^, ^45^ ^]^ Nano CaP, which is similar in size and structure to the natural nano‐hydroxyapatite present in bone tissue, provides obvious benefits in osteogenesis induction.^[^ ^44^, ^63^ ^]^ Therefore, it is reasonable to say that our biomimetic CaP nanosheets not only exhibited superior biocompatibility but also provided excellent osteogenic effects.

### 2.6 In Vivo Antibacterial Evaluation of Nano‐Micro CaP@MS‐Oligo‐Van Composites

**Figure 6:**
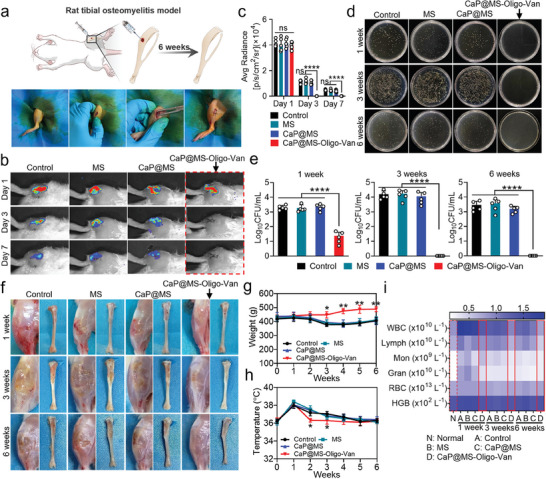
Bactericidal performance of the nano‐micro CaP@MS‐Oligo‐Van composites in an MRSA‐infected rat osteomyelitis model. Luminescent MRSA was used in these experiments. Infected tibias were locally injected with PBS (control), MS, CaP@MS, or CaP@MS‐Oligo‐Van via a minimally invasive approach. The therapeutic effects of the above treatments were recorded and evaluated at weeks 1, 3, and 6. a) Establishment of the rat tibial osteomyelitis model and minimally invasive therapy. b,c) Representative bacterial luminescent images (b), and quantification data (c) from sites of tibial infection after the above treatments. The luminescent images were acquired using IVIS on day 1, 3, and 7 after treatment. d,e) Representative colony plate images of MRSA obtained from the infected bone marrow (d), and the corresponding CFU counts (e) at weeks 1, 3, and 6. f) Representative images of surrounding soft tissue (left) and tibias (right) from infected rats after the above treatments. The pictures were obtained at weeks 1, 3, and 6 after treatment. g,h) The changes in body weight (g) and body temperature (h) after the above treatments. The data were recorded once a week for six continuous weeks. i) Blood routine tests were administered at weeks 1, 3, and 6 after the above treatments. Images are representative of five independent experiments. The results in Figure 6c,e,g,h are presented as the mean ± SD of five independent experiments. ^*^ *p* < 0.05, ^**^ *p* < 0.01, ^****^ *p* < 0.0001. ns, no significance.

Rat models of tibial osteomyelitis infection were used to investigate the antibacterial activity of nano‐micro CaP@MS‐Oligo‐Van composites in vivo. The right tibias of Sprague–Dawley (SD) rats (12 weeks old, males) were drilled (1 mm) and injected with 50 µL of bioluminescent MRSA (10^6^ CFU mL^−1^). Treatments were performed on the following day (**Figure** **6**a).^[^ ^64^ ^]^

On the first day after model induction, IVIS revealed bioluminescence in the tibias of each group. There was no significant difference in fluorescence intensity across the groups, proving that the rat osteomyelitis model was successfully established. Fluorescence disappeared completely by day 3 following treatment in the CaP@MS‐Oligo‐Van group. However, it persisted in the remaining groups even at the 7‐day follow‐up (Figure 6b,c). IVIS results revealed prompt control of the infection in the CaP@MS‐Oligo‐Van groups after treatment, while the infection persisted in the remaining groups. To further demonstrate the ability of CaP@MS‐Oligo‐Van to eliminate the bacterial burden in vivo, infected tibial bone marrow tissue was collected from each group at 1, 3, and 6 weeks after treatment. The infected tissue was then cultured on BHI agar plates after isocratic dilution for bacterial counting. The results are shown in Figure 6d,e. The findings were consistent with the results observed on IVIS. Specifically, only a few bacteria were detected in the CaP@MS‐Oligo‐Van group at week 1, but no bacteria were observed at both the 3‐ and 6‐week points.

We observed the infected tibias and surrounding soft tissues in rats at 1, 3, and 6 weeks. As illustrated in Figure 6f, yellow abscesses and bone erosion were observed in the control, MS, and CaP@MS groups. These symptoms were most visible at week 3. However, the tibias in the CaP@MS‐Oligo‐Van group remained normal throughout, which indicated that infection was completely eradicated in this group. Furthermore, the tibia hole had mostly healed by week 3 after treatment with CaP@MS‐Oligo‐Van (Figure 6f). The changes in the body weight of rats were also monitored during treatment (Figure 6g). The body weight of rats in the CaP@MS‐Oligo‐Van group increased slowly. However, the rats in the remaining groups showed significant decreases in body weight during the early stage of treatment, and their body weights gradually recovered only in the later stage of treatment. The changes in the body temperature of the rats were also recorded, as shown in Figure 6h. After treatment, normal body temperature was rapidly restored in the CaP@MS‐Oligo‐Van group, while the other groups exhibited a delayed decline in body temperature. Blood analysis at weeks 1, 3, and 6 (Figure 6i) revealed that the counts of white blood cells (WBC), lymphocytes (Lym), monocytes (Mon), and neutrophils (Gran) were closest to normal in the CaP@MS‐Oligo‐Van group. This indicated that the inflammatory response had been significantly alleviated in this treatment group.

**Figure 7:**
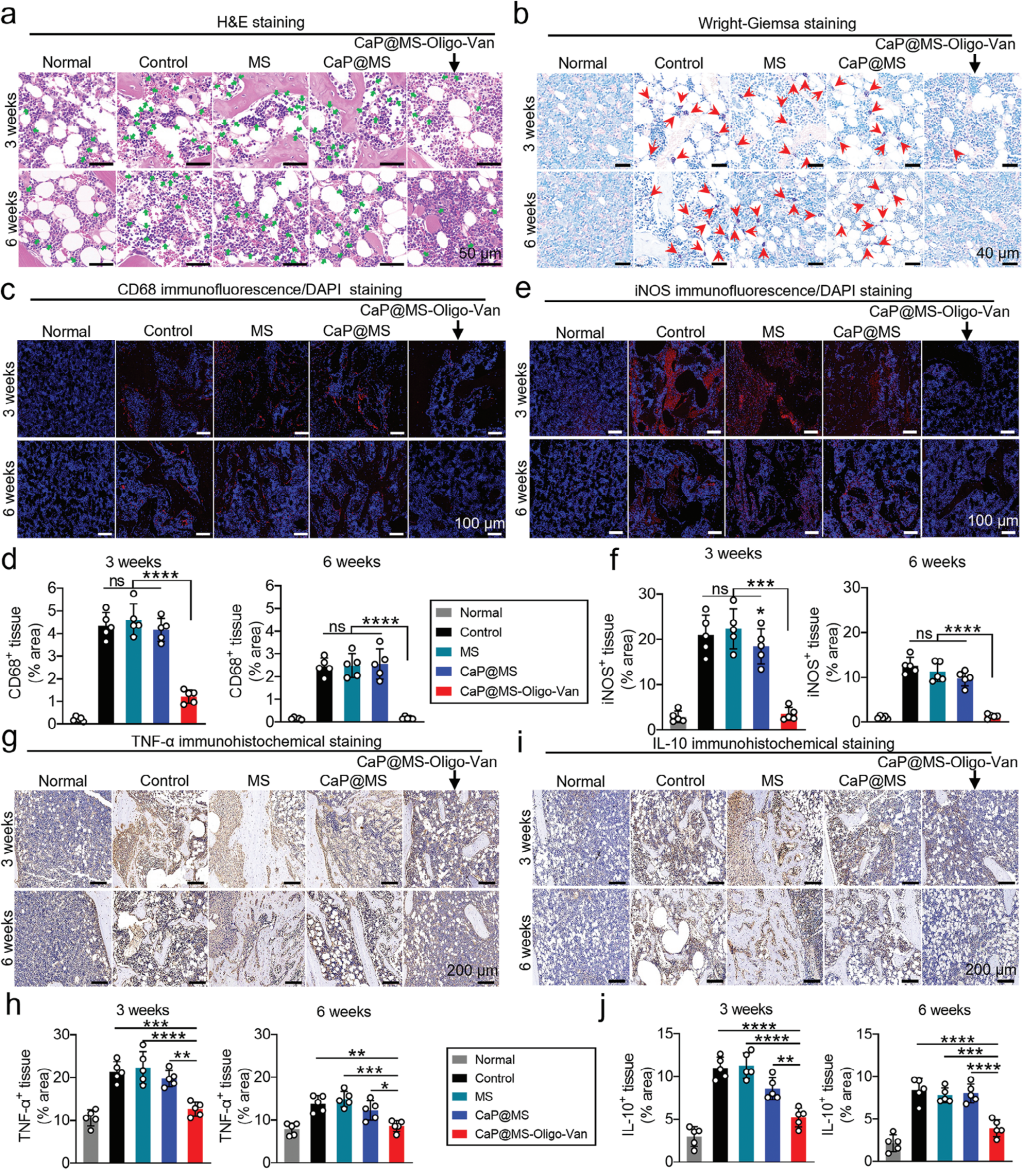
The nano‐micro CaP@MS‐Oligo‐Van composites effectively alleviated inflammation in an MRSA‐induced rat osteomyelitis model. The infected tibias were treated with PBS (control), MS, CaP@MS, or CaP@MS‐Oligo‐Van. The anti‐inflammatory effects of the above treatments on tibial osteomyelitis were assessed at week 3 and 6 after treatment. a,b) Representative H&E staining images (a) and Wright‐Giemsa staining images (b) of the infected tibias after the above treatments. c,d) Representative CD68 immunofluorescence/DAPI double‐staining images of infected rat tibias (c), and quantification of the CD68^+^ tissue area (d) at weeks 3 and 6 after the above treatments. e,f) Representative iNOS immunofluorescence/DAPI double‐staining images of infected rat tibias (e), and quantification of the iNOS^+^ tissue area (f) at weeks 3 and 6 after the above treatments. g,h) Representative TNF‐α immunohistochemical staining images of infected rat tibias (g), and quantification of the TNF‐α^+^ tissue area at week 3 and 6 after the above treatments. i,j) Representative IL‐10 immunohistochemical staining images of infected rat tibias, and quantification of the IL‐10^+^ tissue area at weeks 3 and 6 after the above treatments. Images are representative of five independent experiments. The results in Figure 7d,f,h,j are presented as the mean ± SD of five independent experiments. ^*^ *p* < 0.05, ^**^ *p* < 0.01, ^***^ *p* < 0.001, ^****^ *p* < 0.0001. ns, no significance.

Hematoxylin–eosin (H&E), Wright‐Giemsa, and immunofluorescence staining were used to assess the degree of tibial infection in each group of rats. H&E staining at 3 and 6 weeks (**Figure** **7**a) demonstrated that the neutrophils in the CaP@MS‐Oligo‐Van group were comparable to those in the control group. In contrast, neutrophil levels were significantly higher in the other groups (green arrows). Wright‐Giemsa staining (Figure 7b) also revealed the presence of considerable bacteria (red arrows) in the bone tissue of the control, MS, and CaP@MS groups. However, minimal bacteria were detected in the CaP@MS‐Oligo‐Van group at week 3, and no significant bacteria were detected at week 6 in this group. CD68 is a pan‐macrophage marker that is upregulated following bone tissue infection and is commonly used to evaluate local immune responses.^[^ ^65^, ^66^ ^]^ As indicated in Figures 7c,d, CD68^+^ macrophage infiltration dropped dramatically at week 3 in the CaP@MS‐Oligo‐Van group and was comparable to that in the normal group after week 6. Immunofluorescence and immunohistochemistry staining were used to further study the inflammatory effect of CaP@MS‐Oligo‐Van in MRSA‐infected osteomyelitis (Figure 7e–j). The levels of inflammatory indicators such as iNOS (Figure 7e,f), TNF‐α (Figure 7g,h), and IL‐10 (Figure 7i,j) were significantly elevated in the control, MS, and CaP@MS groups. However, they were significantly reduced in the CaP@MS‐Oligo‐Van group, approaching normal tissue levels. It is common to see that IL‐10 shows a transient decrease in the early stage of osteomyelitis and then gradually increases subsequently.^[^ ^67^ ^]^ In the immune microenvironment of osteomyelitis, monocytic cells can upregulate Toll‐like receptor 3 (TLR3) gene expression in response to the internalized *S. aureus* or its extracellular vehicles (EVs), then activate the NF‐κB pathway, and promote significant secretion of IL‐10.^[^ ^68^ ^]^ Admittedly, IL‐10 is considered an important M2 marker, which acts as an anti‐inflammatory cytokine.^[^ ^69^ ^]^ While, once the infection was completely eradicated by Van, the number of immune cells, including macrophages, gradually subside and their produced cytokines is correspondingly reduced. Overall, the results indicated that CaP@MS‐Oligo‐Van could effectively eliminate MRSA from tissues and reduce inflammation in local lesions.

### 2.7 In Vivo Bone Regeneration by Nano‐Micro CaP@MS‐Oligo‐Van Composites

**Figure 8:**
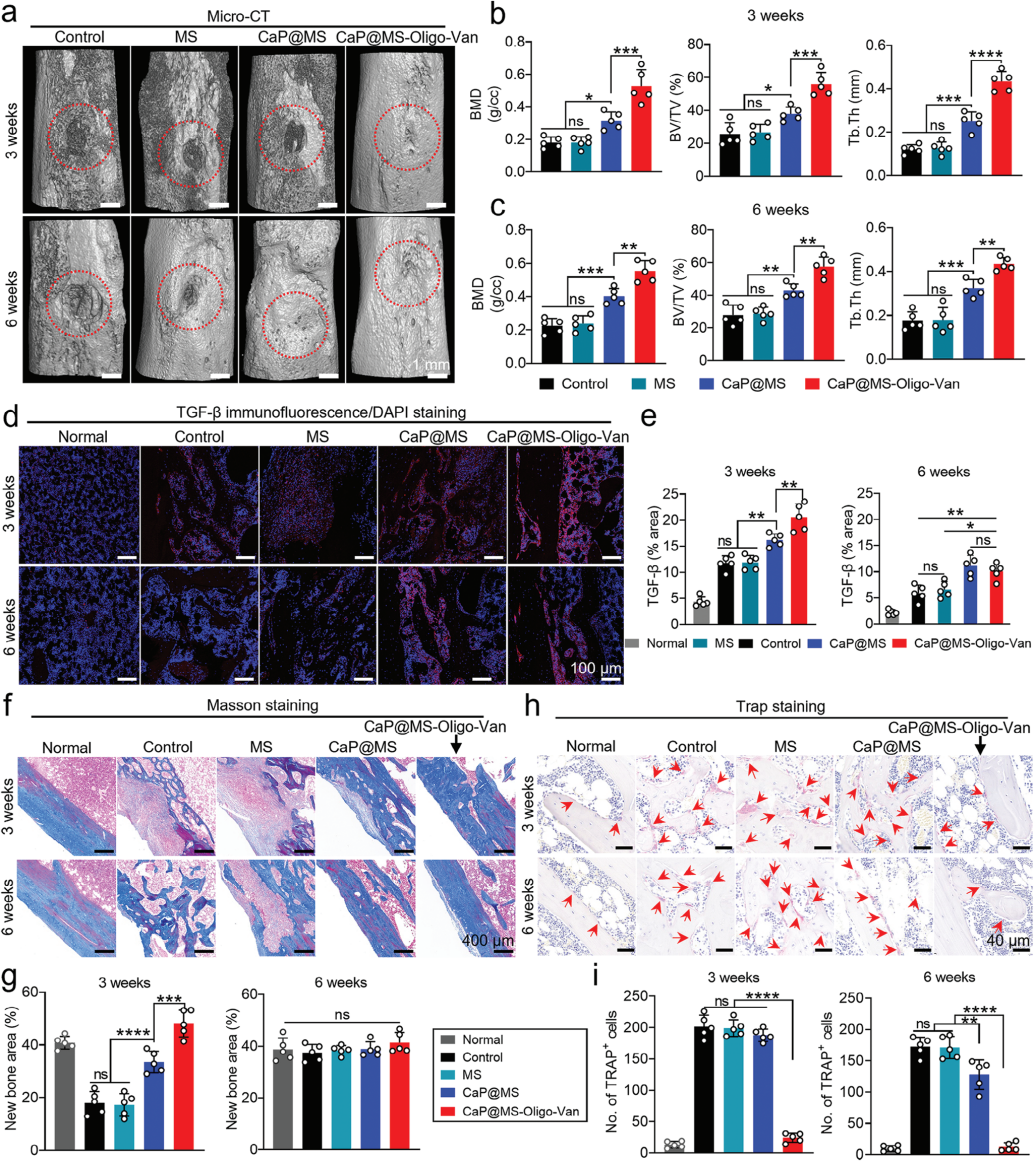
The nano‐micro CaP@MS‐Oligo‐Van composites rapidly promoted bone regeneration in an MRSA‐induced rat osteomyelitis model. MRSA‐infected tibias were locally injected with PBS (control), MS, CaP@MS, or CaP@MS‐Oligo‐Van via minimally invasive surgery. The effects of the above treatments on bone regeneration were evaluated at weeks 3 and 6 after treatment. a) Representative micro‐CT images of the rat tibias. The images were taken at weeks 3 and 6 after the above treatments. b,c) Quantitative analysis of microscopic CT parameters (BMD, BV/TV, and Tb.Th) obtained from Figure 8a.d,e) Representative TGF‐β immunofluorescence/DAPI double‐staining images of the rat tibias (d), and quantification of the TGF‐β^+^ tissue area (e) at weeks 3 and 6 after the above treatments. f,g) Representative Masson staining images of the rat tibias (f), and quantification of the new bone area (g) at weeks 3 and 6 after the above treatment. h,i) Representative TRAP staining images of the rat tibias (h), and quantification of TRAP^+^ cells (i) at weeks 3 and 6 after the above treatments. Images are representative of five independent experiments. The results in Figure b,c,e,g,i are presented as the mean ± SD of five independent experiments. ^*^ *p* < 0.05, ^**^ *p* < 0.01, ^***^ *p* < 0.001, ^****^ *p* < 0.0001. ns, no significance.

X‐ray imaging can reveal changes in bone tissue morphology. X‐ray images of infected tibias at weeks 3 and 6 are shown in Figure S19 (Supporting Information). The control and MS groups had an apparent periosteal reaction, bone degradation, and local bone density reduction surrounding the hole. However, the CaP@MS‐Oligo‐Van group did not exhibit any of these phenomena, and the hole completely healed by week 6. Owing to the osteogenic activity of CaP nanosheets, the local bone tissue destruction in the CaP@MS group was between that seen in the aforementioned two groups. We analyzed collected bone tissue using 3D reconstructed Micro‐CT images (**Figure** **8**a). The CaP@MS‐Oligo‐Van group exhibited the most satisfying bone reconstruction results. In contrast, considerable bone damage was observed in both the control and MS groups at weeks 3 and 6. Meanwhile, the CaP@MS group showed significant recovery at week 6 despite the presence of severe bone destruction at week 3. Finally, the CaP@MS‐Oligo‐Van group showed significantly higher bone mineral density (BMD), bone tissue volume/total tissue volume (BV/TV), and bone trabecular thickness (Tb. Th) values than the other groups (Figure 8b,c). Notably, although the infection persisted, the osteogenic effects of the CaP nanosheets reduced the severity of damage to bone tissue in the CaP@MS group.

Studies have shown that CaP materials can regulate the gene expression of relevant inflammatory factors and growth factors, thereby promoting osteogenesis.^[^ ^70^, ^71^, ^72^ ^]^ The results of TGF‐β immunofluorescence staining are shown in Figure 8d,e. TGF‐β expression was significantly higher in the CaP@MS and CaP@MS‐Oligo‐Van groups. The newly formed bone near the hole was observed using Masson staining (Figure 8f,g). At week 3, the area of new bone was considerably higher in the CaP@MS and CaP@MS‐Oligo‐Van groups than in the control and MS groups. Although there was no significant difference in the amount of new bone among the groups at week 6, the new bone tissue in the CaP@MS and CaP@MS‐Oligo‐Van groups was cleanly aligned and more closely resembled the normal bone cortex. *S. aureus* infection can increase the number of activity of osteoclasts in bone tissue. Hence, it can increase osteolysis by affecting the balance between osteoclasts and osteoblasts.^[^ ^73^, ^74^ ^]^ TRAP staining (Figure 8h,i) revealed that osteoclasts returned to normal levels following treatment in the CaP@MS‐Oligo‐Van group, although they showed a significant increase in the other groups. This suggested that the infection had been effectively controlled in the CaP@MS‐Oligo‐Van group, allowing for the reconstruction of bone tissue.

### 2.8 In Vivo Biocompatibility

We injected MS, CaP@MS, and CaP@MS‐Oligo‐Van into the tibias of normal SD rats and continued to feed them for 5 days. Subsequently, we performed H&E staining of the heart, liver, spleen, lungs, and kidneys in each group (Figure S20, Supporting Information). The results showed that none of the composites exerted significant toxicity against the heart, liver, spleen, lungs, or kidneys in SD rats. In addition, blood biochemical analysis on day 5 (Figure S21, Supporting Information) revealed no significant differences in blood biochemical indices such as alanine aminotransferase (ALT), aspartate aminotransferase (AST), creatinine (CREA), and blood urea nitrogen (BUN) among the groups. Therefore, the composites used in this study were confirmed to have a good in vivo biocompatibility profile.

## 3 Conclusion

In summary, we successfully synthesized smart MN‐responsive CaP@MS‐Oligo‐Van composites using microfluidic technology. These composites showed good bactericidal effects against *S. aureus* and MRSA and helped in effectively treating tissue infection. The bacteria were captured owing to the porous structure of the hydrogel microspheres and the formation of hydrogen bonds between Van and the bacteria. The nucleotide sequences in the oligo were sheared in a site‐specific manner by the nuclease secreted by *S. aureus* and MRSA. This resulted in the rapid release of Van, which killed the infection‐causing bacteria. Moreover, CaP nanosheets were slowly released and promoted bone repair. Overall, in this study, CaP@MS‐Oligo‐Van microspheres with a “capture” function were constructed for minimally invasive injection. Moreover, hydrogel microspheres were endowed with active bacterial capture and targeted release abilities. This enhanced their precise antibacterial effects as well as their ability to activate osteoblasts and inhibit osteoclasts. As a result, these integrated, multipurpose microspheres achieved antibacterial action and bone tissue reconstruction. These multifunctional composites could serve as a valuable strategy for the clinical treatment of osteomyelitis.

## 4 Experimental Section

### Materials

Gelatin was purchased from Sigma–Aldrich (Shanghai, China). Methacrylic anhydride (MA), Van, and triethylamine (C_6_H_15_N) were purchased from Adamas (Shanghai, China). Ammonium phosphate dibasic ((NH_4_)_2_HPO_4_, ≥ 99.0%) and calcium nitrate tetrahydrate (Ca(NO_3_)_2_·4H_2_O, ≥ 99.0%) were purchased from Greagent (Shanghai, China). The oligo (5´‐NH_2_‐mC‐mU‐mC‐mG‐T‐T‐mC‐mG‐mU‐mU‐mC‐NH_2_‐3´) was customized by GenScript Biotech Co., Ltd (Nanjing, China).

### CaP Nanosheet Synthesis

A precipitation technique was used to synthesize CaP nanosheets with a calcium‐to‐phosphorus molar ratio of 10:6.^[^ ^75^ ^]^ Phosphorus and calcium ions were supplied by (NH_4_)_2_HPO_4_ and Ca (NO_3_)_2_·4H_2_O, respectively. First, a solution of 0.15 m (NH_4_)_2_HPO_4_ was prepared, and its pH was adjusted to 10 using NH_3_·H_2_O. The (NH_4_)_2_HPO_4_ solution was then mixed with an equal volume of a 0.25 m Ca (NO_3_)_2_·4H_2_O solution for 2 h using a magnetic stirrer. Anhydrous ethanol was used to wash the precipitate thrice after centrifugation. The precipitate was then dried for a total of 24 h at 60 °C under vacuum.

### Preparation of GelMA Hydrogels

PBS was used for preparing a 10% gelatin solution, which was heated and stirred in an oil bath at 50 °C until the gelatin dissolved completely. MA was added to the gelatin at a rate of 0.2 mL min^−1^, and the reaction was continued for 3 h in the oil bath. Then, 100 mL of PBS was added to stop the reaction. The unreacted MA was removed after centrifugation at 7000 rpm for 15 min, followed by dialysis for 2 days at 38 °C. The final product was obtained via vacuum freeze‐drying.

### Synthesis of Nano‐Micro CaP@MS‐Oligo‐Van Composites

Microfluidic technology was utilized to synthesize CaP@MS.^[^ ^76^ ^]^ Deionized water was used to prepare a 5% GelMA solution. Then, 0.1% CaP nanosheets and 0.2% photo‐initiator were added to this solution and thoroughly mixed while ensuring that the mixture was protected from light. The oil phase consisted of mineral oil, and 5% Span 80 was also included. CaP@MS droplets were generated as a result of the oil phase continually intersecting with the aqueous phase. Photo‐crosslinking was completed after exposure to ultraviolet (365 nm, 10 min) radiation. Subsequently, CaP@MS was recovered and repeatedly cleaned with ether, followed by deionized water. Subsequently, the samples were collected via vacuum freeze–drying. Van was covalently attached to CaP@MS through EDC/NHS chemistry. First, 20 mg CaP@MS and 30 nm Oligo were added to the EDC/NHS reaction solution for 12 h at room temperature. Then, 20 mg Van was added to continue the reaction for 12 h. The reacted microspheres underwent 24‐h dialysis in deionized water. After a second cycle of vacuum freeze–drying, the CaP@MS‐Oligo‐Van samples were collected.

### Characterization of Materials

The morphology and microstructure of the materials were examined using scanning electron microscopy (SEM, S4800, Japan) and transmission electron microscopy (TEM, F200X, USA). An energy‐dispersive spectrometer (EDS, AZtecWave, Britain) was used to obtain elemental mapping images, and X‐ray photoelectron spectroscopy (XPS, NEXSA, USA) was used to analyze the elemental material composition. The chemical structure of the materials was analyzed by Fourier transform infrared (FT‐IR, Nicolet NEXUS 870, USA) spectroscopy. The shape and particle size of nano‐micro CaP@MS‐Oligo‐Van composites were observed using a phase‐contrast optical microscope (PCOM, Nikon, Japan). The pore size distribution and porosity of nano‐micro CaP@MS‐Oligo‐Van composites were examined using a mercury porosimeter (MIP, V9620, USA).

### Drug Loading and Release

The samples were assessed using thermogravimetric analysis (TGA, 8000, USA) at 10 °C min^−1^ in air to estimate the amount of CaP nanosheets loaded into the composites. A UV‐3600i spectrophotometer (SHIMADZU, Japan) was used to measure the loading rate of Van. At first, a calibration curve was generated using five standard Van solutions with concentrations of 10, 20, 30, 40, 50, and 100 µg mL^−1^. The absorbance of the supernatant and dialysate at 280 nm was measured following the synthesis of CaP@MS‐Oligo‐Van to calculate the amount of unreacted Van and indirectly quantify the amount of loaded Van.^[^ ^77^ ^]^ Drug loading (DL) was determined using the following equation:

(1) $$\begin{array}{c} DL=\frac{\text{Total amount of drug}-\text{Amount of drug in supernatant and dialysate}}{\text{Amount of composites}}\;\;\; \end{array}$$

Before testing Van release, the cleavage of the oligo by MN before its binding to Van or GelMA hydrogels via gel permeation chromatography (GPC, LC‐20AD XR, Japan) and UV detection at 260 nm was verified.^[^ ^27^ ^]^ The responsive release of Van from the oligo of CaP@MS‐Oligo‐Van (1 mg mL^−1^) in Ca^2+^‐free PBS (pH 7.4), PBS (pH 7.4) containing MN (0.1 U µL^−1^), and PBS (pH 7.4) containing physiological concentrations of Ca^2+^ and MN (0.1 U µL^−1^) was measured by a UV‐3600i spectrophotometer (280 nm) at different time points.

### Biocompatibility Assessment

A CCK‐8 test kit (Adamas life, QDY‐003‐C, China) was used to examine the toxicity of composites against HUVECs and BMSCs. BMSCs and HUVECs (1 × 10^4^ cells per well) were cultured in plates and co‐incubated with composites (1 mg mL^−1^) for 1, 3, and 5 days. At different time points, an appropriate amount of CCK‐8 reagent was added to each well of the plate, and co‐incubation was continued for 1 h at 37 °C. Then, the OD value (450 nm) was measured using a microplate reader. At the same time, the cell culture solution was gently aspirated, and the cells were stained using the Live/Dead Cell Staining Kit (KeyGEN, KGAF001, China). The pre‐configured working staining solution was added to each well for 15 min at 37 °C, and the cells were observed and evaluated under a fluorescence microscope (Nikon, Japan).

The composites and BMSCs were co‐incubated for 1, 3, and 5 days. Subsequently, the microspheres were washed twice with PBS (pH 7.4) and then fixed with 4% paraformaldehyde at room temperature for 20 min. The microspheres were treated with 0.5% (v/v) Triton X‐100 solution for 10 min and then incubated with blocking buffer for 1 h, before two washes with PBS (pH 7.4). Finally, the cells on the composites were stained with phalloidin (Adamas life, C8082, China) for 12 h and DAPI (Adamas life, C8055, China) for 10 min before observation under a confocal laser scanning microscope (Leica, Germany).

### Hemolysis Test

The hemolysis test was conducted as described previously.^[^ ^55^, ^78^ ^]^ To obtain pure red blood cells (RBCs), 1 mL of rat blood was collected, centrifuged at 1000 rpm for 5 min, and then washed five times with saline. The 4% RBC solution was incubated with the different types of microspheres (1 mg mL^−1^), water, or saline for 12 h at 37 °C. Following another round of centrifugation, the supernatant was obtained and its absorbance at 542 nm was measured using a microplate reader. The hemolysis rate was calculated using the following formula:

(2) $$\mathrm{Hemolysis}\mathrm{rate}\left(\%\right)=\left(I_{542\;\mathrm{nm}/\mathrm{sample}}/I_{542\;\mathrm{nm}/\mathrm{control}}\right)\times 100\%$$

### Antibacterial Test and Bacterial Morphology

The standard plate count method, live/dead staining, and growth curves were used to examine the inhibitory and antibacterial effects of the composites against *S. aureus* and MRSA.^[^ ^79^, ^80^ ^]^ For standard plate counts, each type of microsphere (1 mg mL^−1^) was added to the bacterial suspension (1 × 10^6^ CFU mL^−1^) containing brain heart infusion (BHI) broth and incubated for 6 h at 37 °C. The suspension was then serially diluted and inoculated onto BHI agar plates. This was followed by incubation at 37 °C for 12 h and the evaluation of CFU counts. For live/dead staining, the bacterial suspension (1 × 10^6^ CFU mL^−1^) was incubated with each type of microsphere (1 mg mL^−1^) for 2 h at 37 °C. Subsequently, the bacteria were stained for 15 min with the SYTO 9/PI Live/Dead Bacterial Double Stain Kit (MKBio, China) and then observed under a fluorescence microscope. ImageJ software was used to calculate the proportion of dead bacteria. Additionally, the bacteria and microspheres were co‐incubated for 2 h, fixed with glutaraldehyde for 2 h, and then successively dehydrated with a gradient of ethanol solutions. Subsequently, the samples were air‐dried and the morphology of the bacteria was observed using SEM.

Bacteria (5 × 10^5^ CFU mL^−1^) were also co‐incubated with each type of microsphere (1 mg mL^−1^) at 37 °C for 24 h to conduct growth curve analysis. A microplate reader was used to detect the absorbance at 600 nm at the appropriate time intervals.

### Bacterial Capture

Bacteria (1 × 10^6^ CFU mL^−1^) and microspheres (1 mg mL^−1^) were co‐incubated in PBS and placed in a bacterial shaker at 37 °C for 30 min. The supernatant and bacteria present on the microspheres were separated for plate counting to calculate the number of CFUs. The bacteria on the microspheres were separated via shaking with ultrasound (1 W cm^−2^) and allowed to resuspend in the same volume of PBS (pH 7.4). Bacteria (1 × 10^6^ CFU mL^−1^) and microspheres (1 mg mL^−1^) were co‐incubated in BHI broth and placed in a bacterial shaker at 37 °C for 12 h. To calculate CFU counts, the bacteria in the supernatant and on the microspheres were coated as described previously. Furthermore, the bacteria on the microspheres were examined using live/dead staining, observed with a confocal laser scanning microscope, and fluorescently quantified with ImageJ software.

After co‐incubation with bacteria for 12 h, the microspheres were collected, fixed with pentanediol for 2 h, and dehydrated with different concentrations of ethanol. The samples were air‐dried, and finally, the morphology of the bacteria caught on the microspheres was observed using SEM.

### Alizarin Red S Staining and Alkaline Phosphatase (ALP) Staining

BMSCs were grown for 24 h at a density of 1 × 10^4^ cells per well in 24‐well plates. The medium was aspirated after 24 h, and osteogenic induction solution (β‐Glycerophosphate: 10 mm, Ascorbic Acid: 300 nm, Dexamethasone: 10 nm) containing 1 mg mL^−1^ of composites was added. This solution was then changed every 2 days. The mineralized nodules were stained with alizarin red (1%, pH 8.0) after 14 days of incubation and examined under a light microscope. After decolorizing the mineralized nodules with cetylpyridinium chloride (Sigma–Aldrich, USA) for 15 min, OD values were measured at 562 nm using a microplate reader.^[^ ^81^ ^]^

On day 7, the cells were stained using an Alkaline Phosphatase Color Development Kit (Beyotime, C3206, China), and images were obtained under a light microscope. ALP activity was also detected using the Alkaline Phosphatase Assay Kit (Beyotime, P0321S, China).^[^ ^81^ ^]^

### Quantitative Real‐Time Polymerase Chain Reaction (qRT–PCR) and Western Blot

The expression levels of osteogenic genes encoding alkaline phosphatase (ALP), Runt‐related transcription factor 2 (RUNX2), Type I collagen (COL‐1), and osteocalcin (OCN) in BMSCs were measured via qRT‐PCR. BMSCs were seeded on a 6‐well culture plate at a density of 1 × 10^4^ per well, and 2 mL of osteogenic induction medium and 2 mg of composites were added to each well. Every 2 days, the medium was changed. The expression of osteogenesis‐related genes was examined after 14 days of culture. Total RNA was extracted using TRIzol reagent (Invitrogen, USA), followed by reverse transcription with the Prime Script RT‐PCR Kit (TAKARA, Japan). Finally, qRT‐PCR was carried out using the SYBR Green RT‐PCR Kit (Qiagen, Germany) on the CFX Connect Real‐Time PCR Detection System (Bio‐Rad, USA). The primer sequences for each gene are shown in Table S2 (Supporting Information).

Weston blot was used to detect the expression of the osteogenesis‐related proteins ALP, RUNX2, COL‐1, and OCN. After 14 days of co‐incubation between BMSCs and composites, the cells were lysed in lysis buffer (Adamas life, China) containing proteinase inhibitor (Sigma–Aldrich, USA) on an ice bath. The total protein content was determined using a BCA protein assay (Adamas life, China). Then, 40 µg of protein was separated using sodium dodecyl sulfate‐polyacrylamide gel (Sigma–Aldrich, USA) electrophoresis. The separated proteins were then transferred to polyvinylidene fluoride (PVDF) membranes. The membranes were treated with 5% bovine serum albumin (BSA; Sigma–Aldrich, USA) for 2 h before incubation with primary antibodies overnight at 4 °C (anti‐ALP: GB113300, Servicebio, 1:1000; anti‐RUNX2: GB13264, Servicebio, 1:1000; anti‐COL‐1: GB114197, Servicebio, 1:1000; anti‐OCN: DF12303, Servicebio, 1:1000). This was followed by incubation with anti‐rabbit or anti‐mouse horseradish peroxidase (HRP)‐conjugated secondary antibodies (Servicebio, 1:5000) at room temperature for 1 h. Finally, blots were detected and imaged using a chemiluminescence instrument (CLINX, 6100, China). ImageJ software was applied to quantify the density of protein expression in each group.

### Establishment of Rat Osteomyelitis Model and Treatment

The experimental animals used in this study were 12‐week‐old male SD rats with an average weight of 428.2 ± 21.19 g. The rats were purchased from Shanghai Chilie Biotechnology Co., Ltd. (Shanghai, China). All animal experiments were performed in compliance with the Institutional Animal Care and Use Committee guidelines of Shanghai Jiao Tong University (protocol number: 2021022401). The procedures strictly adhered to the guidelines provided by the Ministry of Health of the People's Republic of China. The rats were randomized to the following five groups: normal group, control group, MS group, CaP@MS group, and CaP@MS‐Oligo‐Van group. Each group contained 15 rats.

Following isoflurane (RWD, China) anesthesia, the surgical site was disinfected and covered with a surgical hole towel. Then, an incision of ≈1 cm was made in the anteromedial upper 1/3rd of the right tibia to reveal the tibial bone surface. A 1‐mm diameter bone defect was drilled using a medical electric drill. Then, 50 µL of MRSA solution (1 × 10^6^ CFU mL^−1^) was injected into the bone marrow cavity. The hole was filled with bone wax and the surgical site was sutured layer by layer. After 1 day of bone tissue infection, the surgical incision was opened again. The control group received an injection containing 50 µL saline. Meanwhile, the MS, CaP@MS, and CaP@MS‐Oligo‐Van groups received injections containing 50 µL of the corresponding composites dispersed in saline (1 mg mL^−1^). After treatment, the rats were fed regularly for an additional 6 weeks.

### General Condition of the Animals and Hematological Analysis

Before the establishment of the osteomyelitis model, the initial body weight and body temperature of each group of rats were determined. The body weight and body temperature were then routinely monitored once a week for 6 weeks following treatment. Venous blood was obtained from the rats at weeks 1, 3, and 6 following treatment. Standard blood tests were performed to assess the level of systemic infection in these rats.

### Antibacterial Effects of CaP@MS‐Oligo‐Van in Vivo

On days 1, 3, and 7 after infection, the IVIS imaging system was utilized to observe the fluorescence signal from the bone tissue and assess infection. Infected tibias were removed for microbiological evaluation at weeks 1, 3, and 6 following treatments.^[^ ^82^ ^]^ Bone marrow was obtained from the tibias and then frozen in sterile test tubes. The specimens were promptly placed in 1 mL of sterile physiological saline and homogenized for 5 min. Finally, 0.1 mL of the diluent was spread on a BHI culture plate. The CFU count was calculated after 12 h of incubation at 37 °C.

### Gross Observation and Radiographic Evaluation

At weeks 1, 3, and 6 after treatment, the rats were euthanized under anesthesia. The initial incision was repeated to provide access, and tibial morphology and the condition of adjacent soft tissues were observed. Rats were euthanized at 3 and 6 weeks after treatment. The tibias on the infected side were harvested, preserved in 4% paraformaldehyde solution, and immediately examined via Micro‐CT (Belgium, Bruker, Skyscan 1076; spatial resolution, 18 µm) scanning and 3D reconstruction. For quantitative comparisons, bone mineral density (BMD), bone tissue volume/total tissue volume (BV/TV), and bone trabecular thickness (Tb. Th) were measured.

### Histological Examination

At weeks 3 and 6, rats were euthanized, and their tibias were collected. The tibias were promptly fixed in 4% paraformaldehyde, followed by 6 weeks of decalcification in 10% EDTA. Subsequently, the tissue was subjected to dehydration using an ethanol gradient. Dehydrated tissues were embedded in paraffin, sectioned, and finally stained. To assess inflammation and bacterial infiltration due to osteomyelitis, H&E, and Giemsa staining were performed. Masson and TRAP staining were performed to evaluate bone destruction. Immunohistochemical and immunofluorescence staining was applied to detect inflammation and osteogenesis‐related indicators, including CD68‐labeled macrophages, iNOS, TNF‐*α*, IL‐10, and TGF‐*β*. ImageJ software was used for all semi‐quantitative analyses.

### Statistical Analysis

All experiments were performed in three or more replicates. Results were expressed as the mean ± standard deviation (SD). Statistical analyses were performed using SPSS software (version 21, USA). For intergroup comparisons, repeated data were examined based on variance analysis. Statistical significance was determined according to the following *P* values: ^*^ < 0.05, ^**^ < 0.01, ^***^ < 0.001, and ^****^ < 0.0001. All graphs were prepared using GraphPad Prism (version 9.4.1, USA).

## Conflict of Interest

The authors declare no conflict of interest.

## Author Contributions

G.L., G.Z., and S.W. authors contributed equally to this work. L.M., C.M., Z.X.Y, and D.L.F conceived the idea and provided guidance. L.G.H., Z.G., and W.S. conducted experiments, data analysis, and manuscript writing. S.Q., W.B., and Z.Z.Y. performed the microfluidic study. L.H.Q., Y.Q., and W.L. conducted computational analyses. F.Z. and Y.L.S. conducted the typography of figures. All authors edited or commented on the manuscript.

## Supporting information

Supporting Information

Supplemental Video 1

## Data Availability

The data that support the findings of this study are available from the corresponding author upon reasonable request.
