# Supplementary material for: Injectable Nano‐Micro Composites with Anti‐bacterial and Osteogenic Capabilities for Minimally Invasive Treatment of Osteomyelitis
Source: Adv Sci (Weinh). 2024 Jan 17;11(12):2306964. doi: 10.1002/advs.202306964 (PMC10966557; doi:10.1002/advs.202306964)
Supplement: Supplementary file 1 — Supporting Information [file ADVS-11-2306964-s001.pdf]

ADVANCED SCIENCE
Open Access

Supporting Information

for *Adv. Sci.*, DOI 10.1002/advs.202306964

Injectable Nano-Micro Composites with Anti-bacterial and Osteogenic Capabilities for Minimally Invasive Treatment of Osteomyelitis

Guanghua Lu, Gang Zhao, Shen Wang, Hanqing Li, Qiang Yu, Qi Sun, Bo Wang, Li Wei, Zi Fu, Zhenyu Zhao, Linshan Yang, Lianfu Deng, Xianyou Zheng, Ming Cai* and Min Lu**

Supporting Information

Injectable Nano-Micro Composites with Anti-bacterial and Osteogenic Capabilities for Minimally Invasive Treatment of Osteomyelitis

Guanghua Lu[1,2#], Gang Zhao[2#], Shen Wang[3#], Hanqing Li[2], Qiang Yu[2], Qi Sun[1], Bo Wang[1], Li Wei[2], Zi Fu[2], Zhenyu Zhao[1], Linshan Yang[4], Lianfu Deng[2], Xianyou Zheng[5], Ming Cai[1]*, Min Lu[2]**

G. Lu, Q. Sun, B. Wang, Z. Zhao, M. Cai

[1] Department of Orthopaedics, Shanghai Tenth People's Hospital, Tongji University School of Medicine, Shanghai 200072, P. R. China.

E-mail: cmdoctor@tongji.edu.cn

G. Lu, G. Zhao, H. Li, Q. Yu, L. Wei, Z. Fu, L. Deng, M. Lu

[2] Department of Orthopaedics, Shanghai Key Laboratory for Prevention and Treatment of Bone and Joint Diseases, Shanghai Institute of Traumatology and Orthopedics, Ruijin Hospital, Shanghai Jiao Tong University School of Medicine, Shanghai 200240, P. R. China.

E-mail: lumin111@sjtu.edu.cn

S. Wang

[3] Department of Plastic and Reconstructive Surgery, Shanghai Ninth People's Hospital, Shanghai Jiao Tong University School of Medicine, Shanghai 200011, China.

L. Yang

[4] Taikang Bybo Dental, Shanghai 200001, P. R. China.

X. Zheng

[5] Department of Orthopedic Surgery, Shanghai Sixth People's Hospital Affiliated to Shanghai Jiao Tong University School of Medicine, Shanghai, 200233, China.

E-mail: zhengxianyou@126.com

* Correspondence to Min Lu (lumin111@sjtu.edu.cn), Ming Cai (cmdoctor@tongji.edu.cn), and Xianyou Zheng (zhengxianyou@126.com).

# These authors contributed equally to this work.

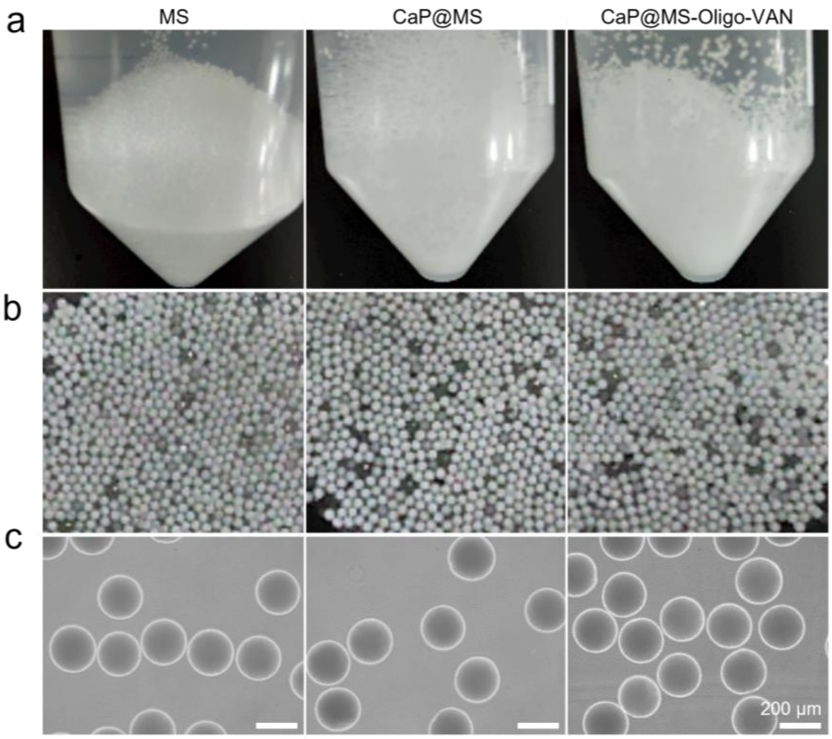

Figure S1. (a, b) Photographs of MS, CaP@MS, and CaP@MS-Oligo-Van. (c) Micrographs of MS, CaP@MS, and CaP@MS-Oligo-Van acquired by optical microscopy. Images are representative of three independent experiments.

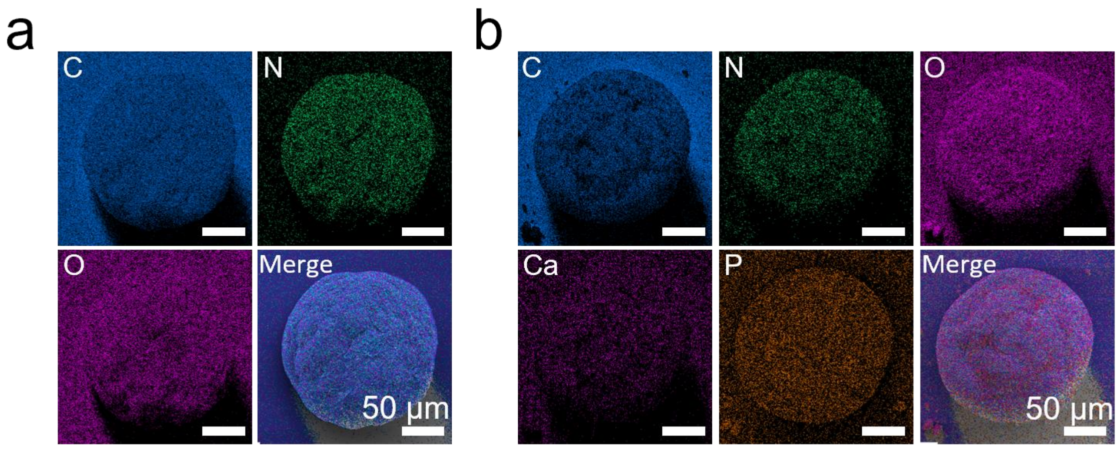

Figure S2. EDS element mapping of MS (a) and CaP@MS (b). Images are representative of three independent experiments.

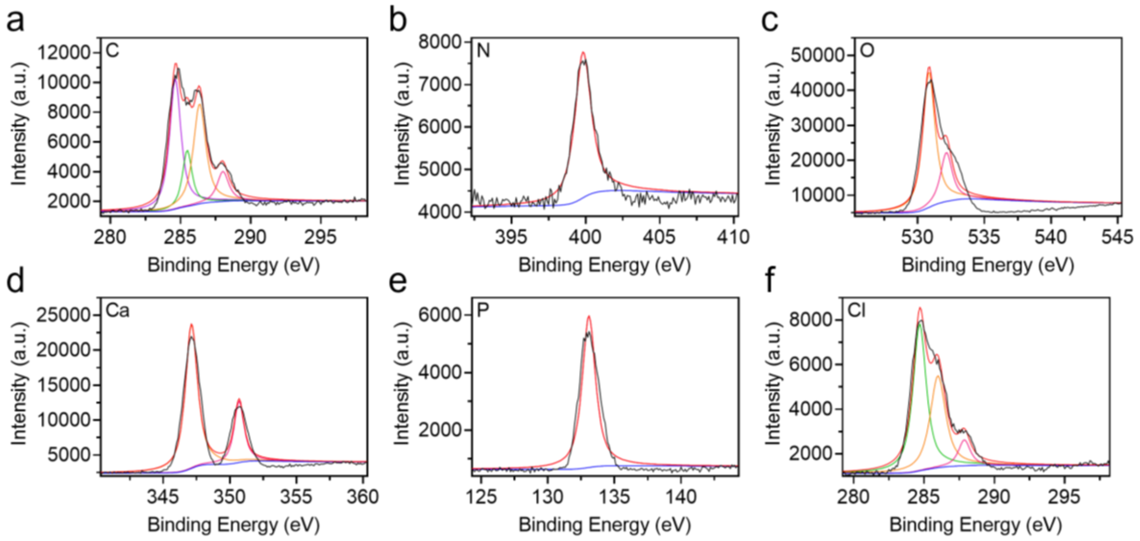

Figure S3. XPS spectrum of the corresponding C (a), N (b), O (c), Ca (d), P (e), and Cl (f) elements in the CaP@MS-Oligo-Van composites. Images are representative of three independent experiments.

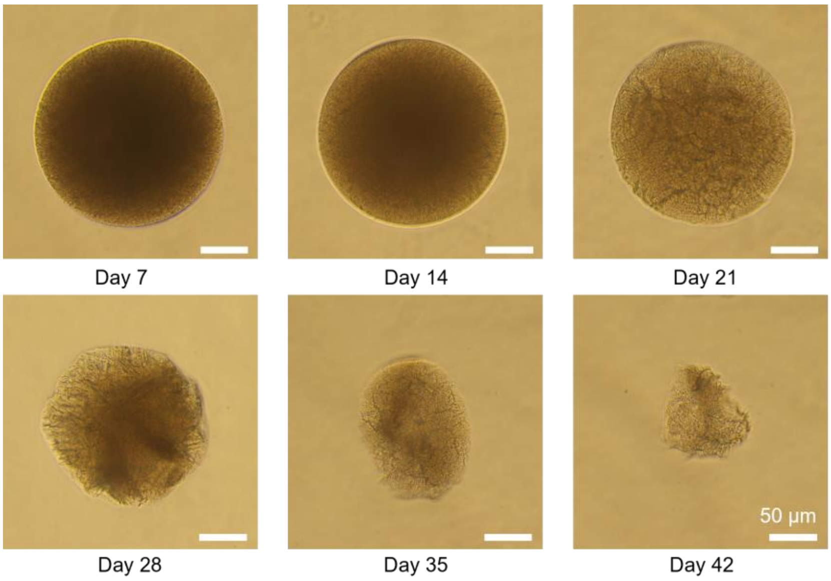

Figure S4. Micrographs of self-degradability of the CaP@MS-Oligo-Van composites in MEM-α medium acquired by optical microscopy. The pictures were taken once a week for 6 continuous weeks. Images are representative of three independent experiments.

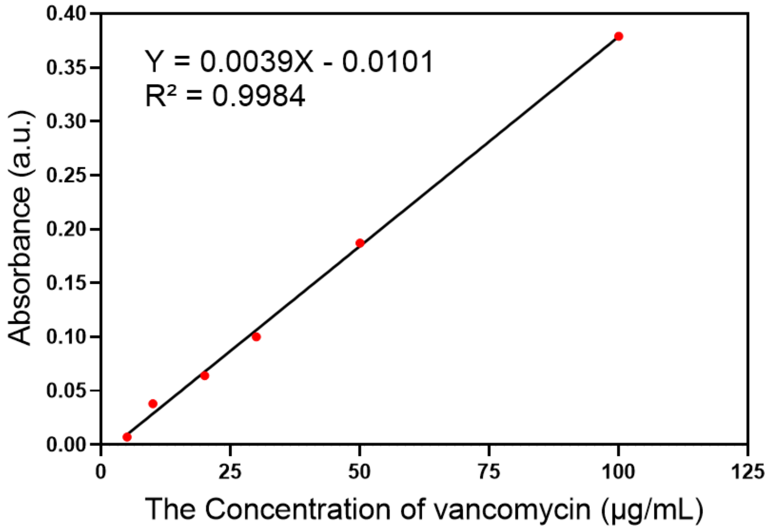

Figure S5. Standard curve of Van. The absorbance at 280 nm of six standard Van solutions with 10, 20, 30, 40, 50, and 100 μg/mL concentrations. Image is representative of three independent experiments.

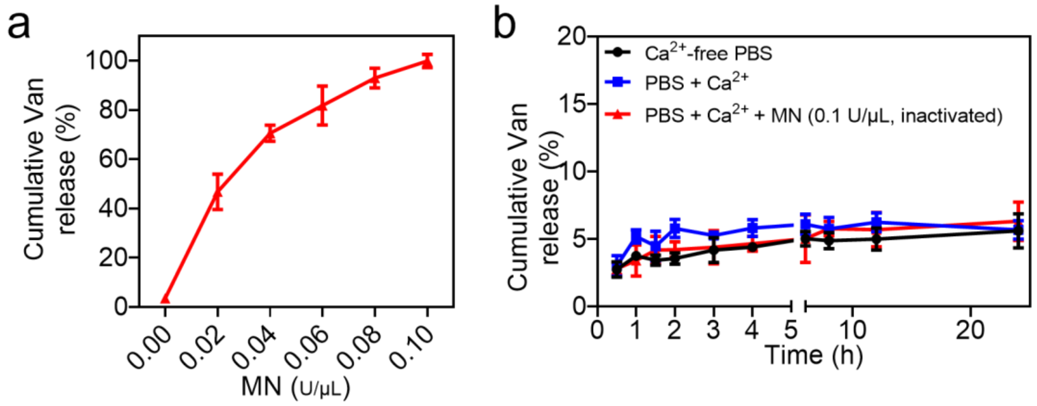

Figure S6. (a) The responsive release of Van from the oligo of CaP@MS-Oligo-Van in PBS (pH 7.4) containing physiological concentrations of Ca^{2+} and different concentrations of MN at 2h. (b) The responsive release of Van from the oligo of CaP@MS-Oligo-Van in Ca^{2+}-free PBS (pH 7.4), PBS (pH 7.4) containing physiological concentrations of Ca^{2+}, and PBS (pH 7.4) containing physiological concentrations of Ca^{2+} and MN (0.1 U/μL, inactivated). The results are presented as means ± SD from five independent experiments.

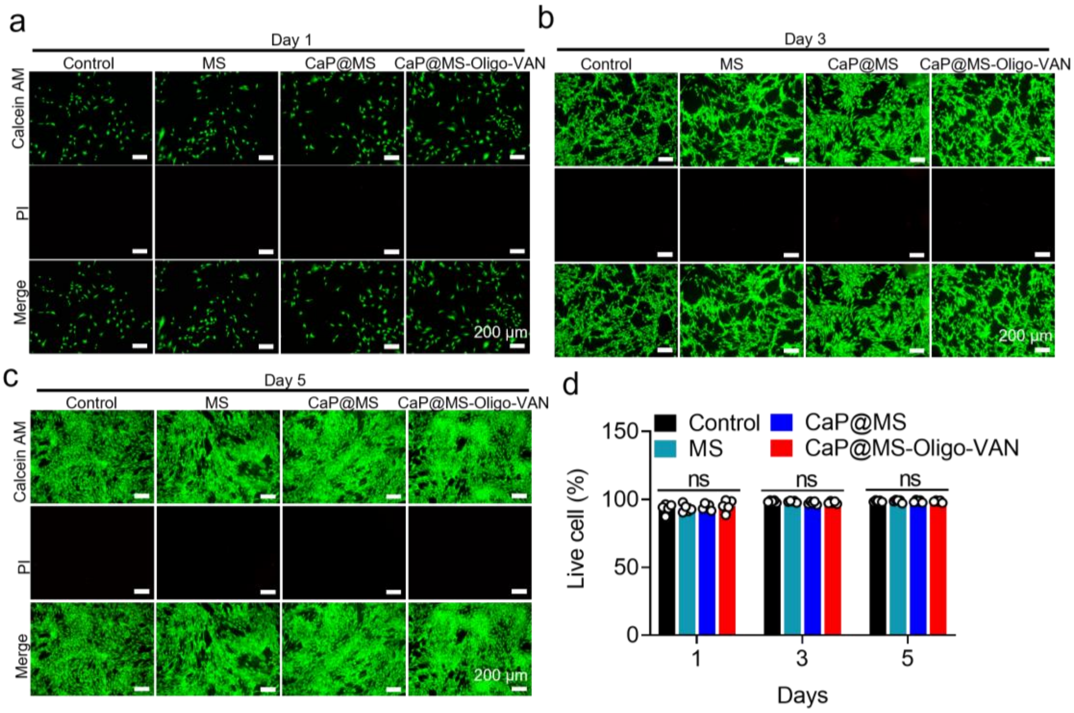

Figure S7. Representative live/dead fluorescence images of BMSCs treated with MS, CaP@MS, and CaP@MS-Oligo-Van. The BMSCs were cocultured with MS, CaP@MS, and CaP@MS-Oligo-VAN for 1 (a), 3 (b), and 5 (c) days, and further processed the live/dead staining, in which the viable and dead cells were visualized by calcein-AM (green) and PI staining (red), respectively. (d) The percentage of calcein-AM stained BMSCs in all cells was statistically calculated by ImageJ software. Images are representative of five independent experiments. The results in Figure S7d are presented as means ± SD from five independent experiments. ns, no significance.

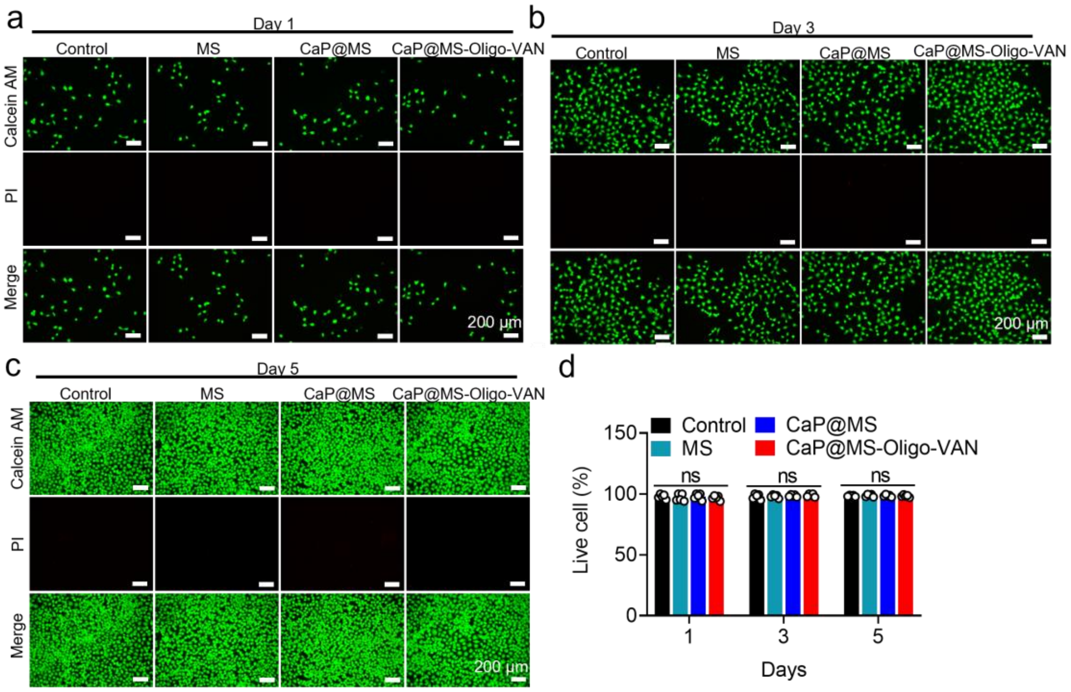

Figure S8. Representative live/dead fluorescence images of HUVEC treated with MS, CaP@MS, and CaP@MS-Oligo-Van. The HUVEC were cocultured with MS, CaP@MS, and CaP@MS-Oligo-VAN for 1 (a), 3 (b), and 5 (c) days, and further processed the live/dead assay, in which the viable and dead cells were visualized by calcein-AM (green) and PI staining (red), respectively. (d) The percentage of calcein-AM stained HUVEC in all cells was statistically calculated by ImageJ software. Images are representative of five independent experiments. The results in Figure S8d are presented as means ± SD from five independent experiments. ns, no significance.

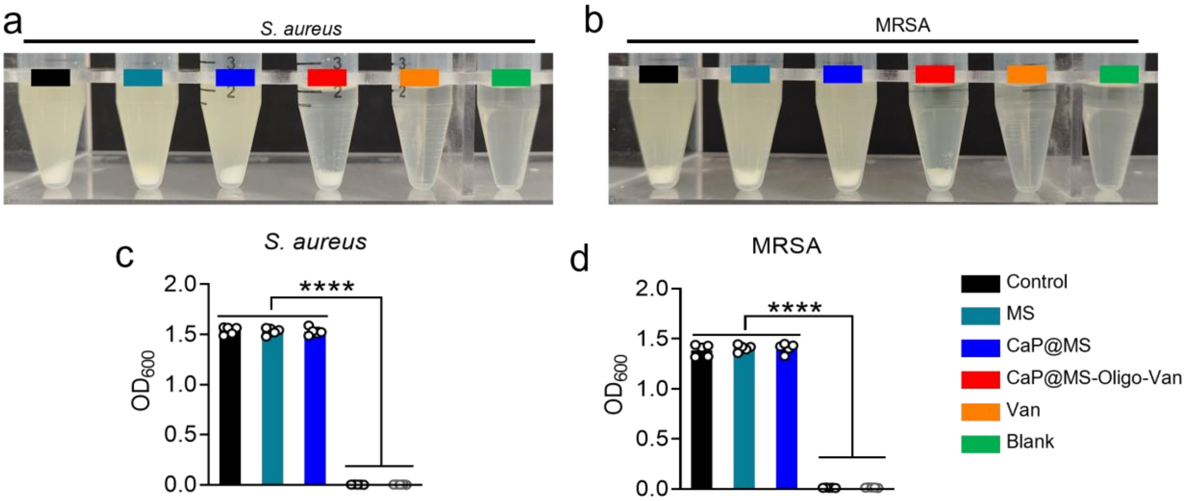

Figure S9. *In vitro* bactericidal effects of the nano-micro CaP@MS-Oligo-Van composites against *S. aureus* (a and b) and MRSA (c and d) in BHI medium. (a and c) Representative photographs of centrifuge tubes contained with *S. aureus* (a) and MRSA (c) treated with MS, CaP@MS, CaP@MS-Oligo-Van, and VAN for 6 h. (b, d) The absorbance of *S. aureus* (b) and MRSA (d) at 600 nm after the above treatments. Images are representative of five independent experiments. The results in Figure S9c and d are presented as means ± SD from five independent experiments. **** $P <$ 0.0001.

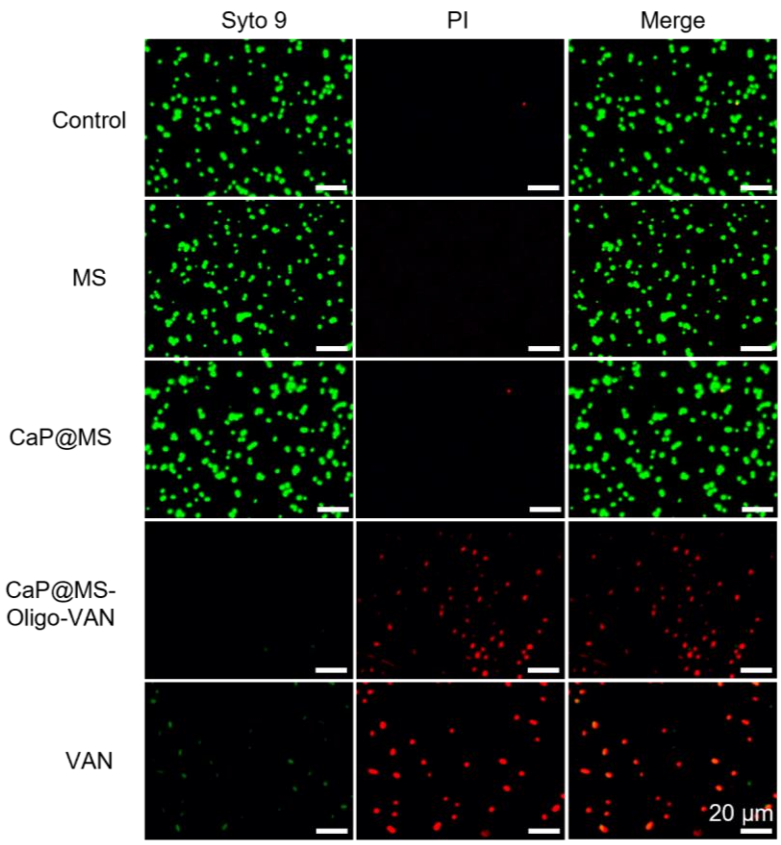

Figure S10. Representative live/dead staining images of *S. aureus* after the PBS (control), MS, CaP@MS, CaP@MS-Oligo-VAN, and VAN alone treatments, in which the visible and dead cells were stained by syto 9 (green) and PI (red), respectively. Images are representative of five independent experiments.

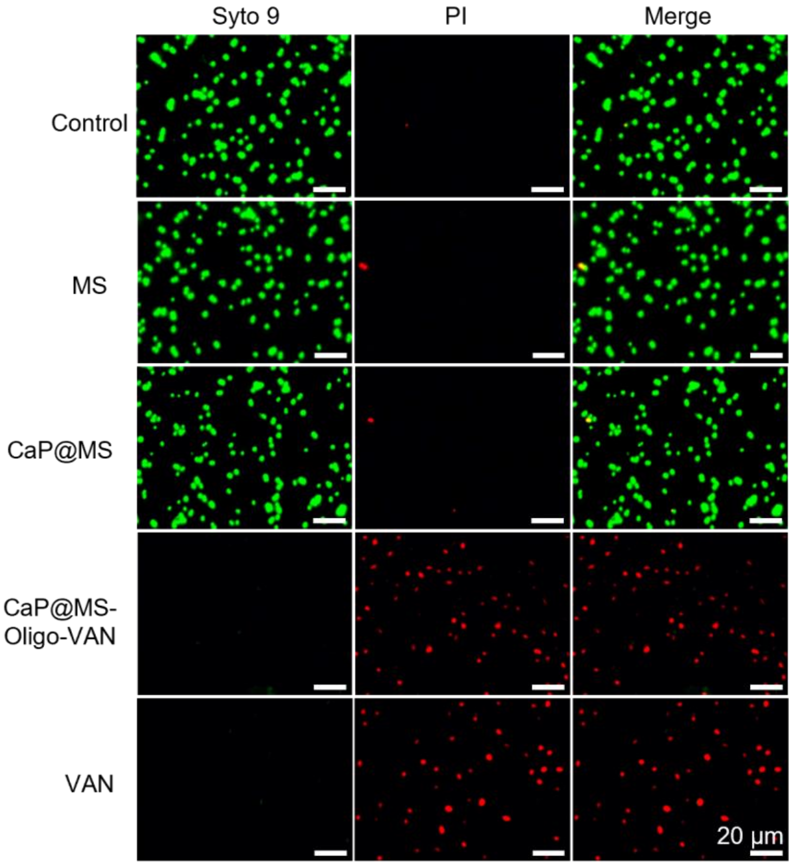

Figure S11. Representative live/dead staining images of MRSA after the PBS (control), MS, CaP@MS, CaP@MS-Oligo-VAN, and VAN alone treatments, in which the visible and dead bacteria were stained by syto 9 (green) and PI (red), respectively. Images are representative of five independent experiments.

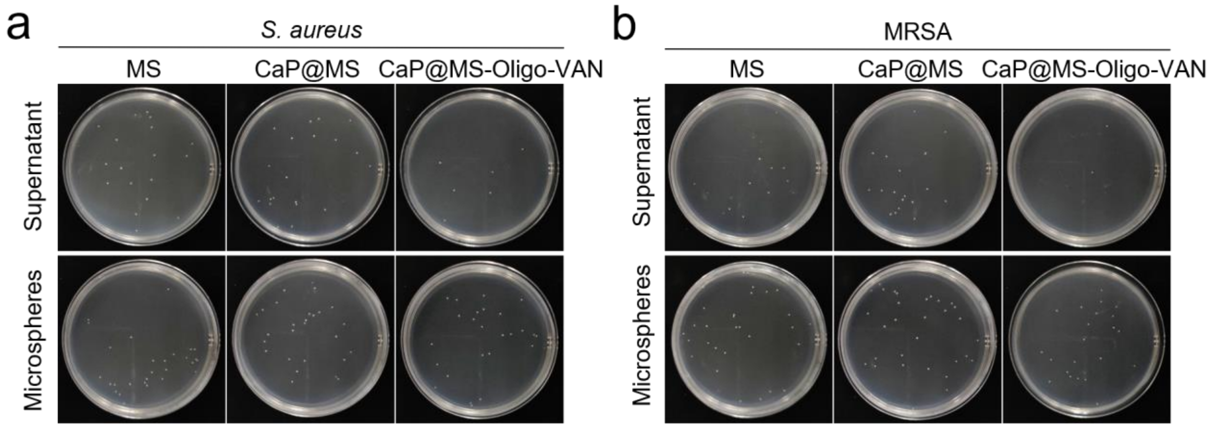

Figure S12. Representative colony plate photographs of *S. aureus* (a) and MRSA (b) from supernatants (upper) and microspheres (down) in PBS. Briefly, *S. aureus* (a) and MRSA (b) were co-incubated with MS, CaP@MS, and CaP@MS-Oligo-Van in PBS for 30 min respectively, then collected the supernatants and microspheres, further cultured for another 24 h on BHI agar plates in 37 °C incubator. Images are representative of five independent experiments.

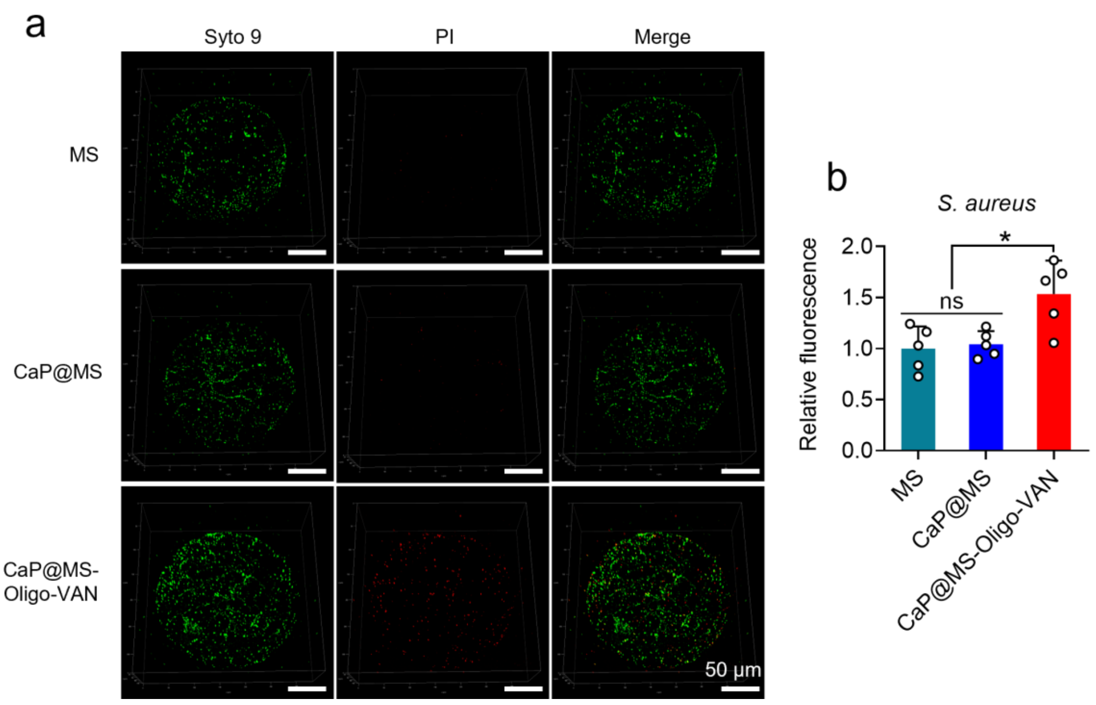

Figure S13. The capture capability of the nano-micro CaP@MS-Oligo-Van composites on *S. aureus* in PBS. (a) Representative fluorescence images of live and dead *S. aureus* absorbed on the surface of MS, CaP@MS, and CaP@MS-Oligo-VAN. The microspheres were collected by centrifuging, stained with syto 9/PI, then further observed by CLSM. The visible and dead cells were stained by syto 9 (green) and PI (red), respectively. (b) Quantification of bacterial fluorescence on the surface of MS, CaP@MS, and CaP@MS-Oligo-VAN. Images are representative of five independent experiments. The results in Figure b are presented as means ± SD from five independent experiments. * $P < 0.05$. ns, no significance.

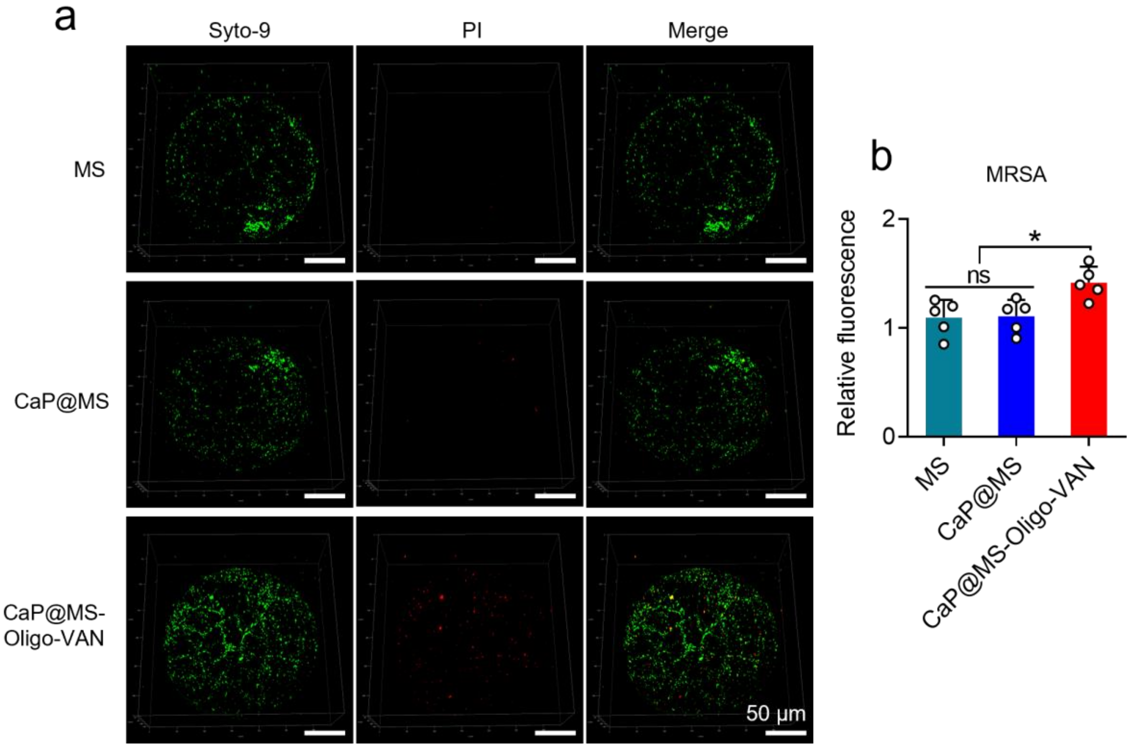

Figure S14. The capture capability of the nano-micro CaP@MS-Oligo-Van composites on MRSA in PBS. (a) Representative fluorescence images of live and dead MRSA absorbed on the surface of MS, CaP@MS, and CaP@MS-Oligo-VAN. The microspheres were collected by centrifuging, stained with syto 9/PI, then further observed by CLSM. The visible and dead cells were stained by syto 9 (green) and PI (red), respectively. (b) Quantification of bacterial fluorescence on the surface of MS, CaP@MS, and CaP@MS-Oligo-VAN. Images are representative of five independent experiments. The results in Figure b are presented as means ± SD from five independent experiments. * $P < 0.05$. ns, no significance.

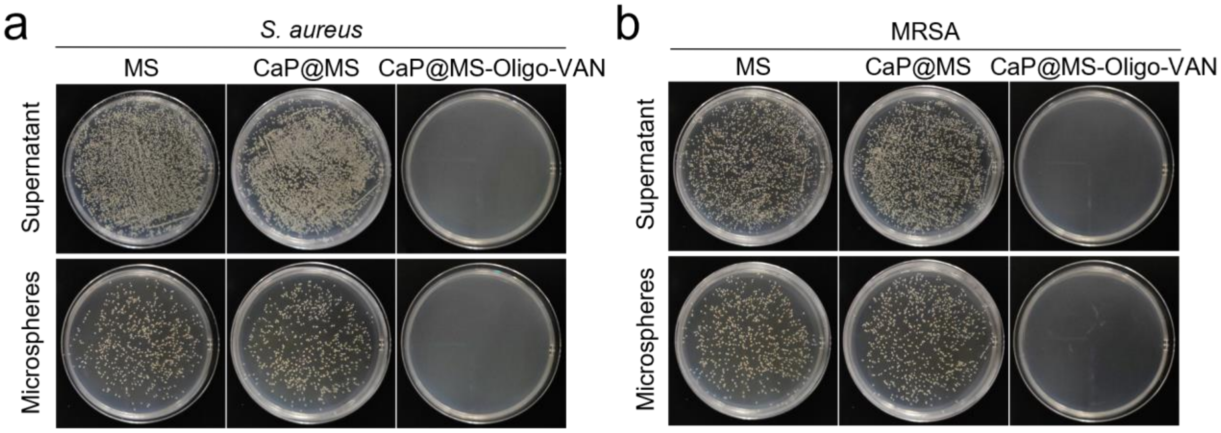

Figure S15. Representative colony plate photographs of *S. aureus* (a) and MRSA (b) from supernatants (upper) and microspheres (down) in BHI medium. Briefly, *S. aureus* (a) and MRSA (b) were co-cultured with MS, CaP@MS, and CaP@MS-Oligo-Van for 6 h in BHI medium respectively, then collected the supernatants and microspheres, further cultured for another 24 h on BHI agar plates in 37 °C incubator. Images are representative of five independent experiments.

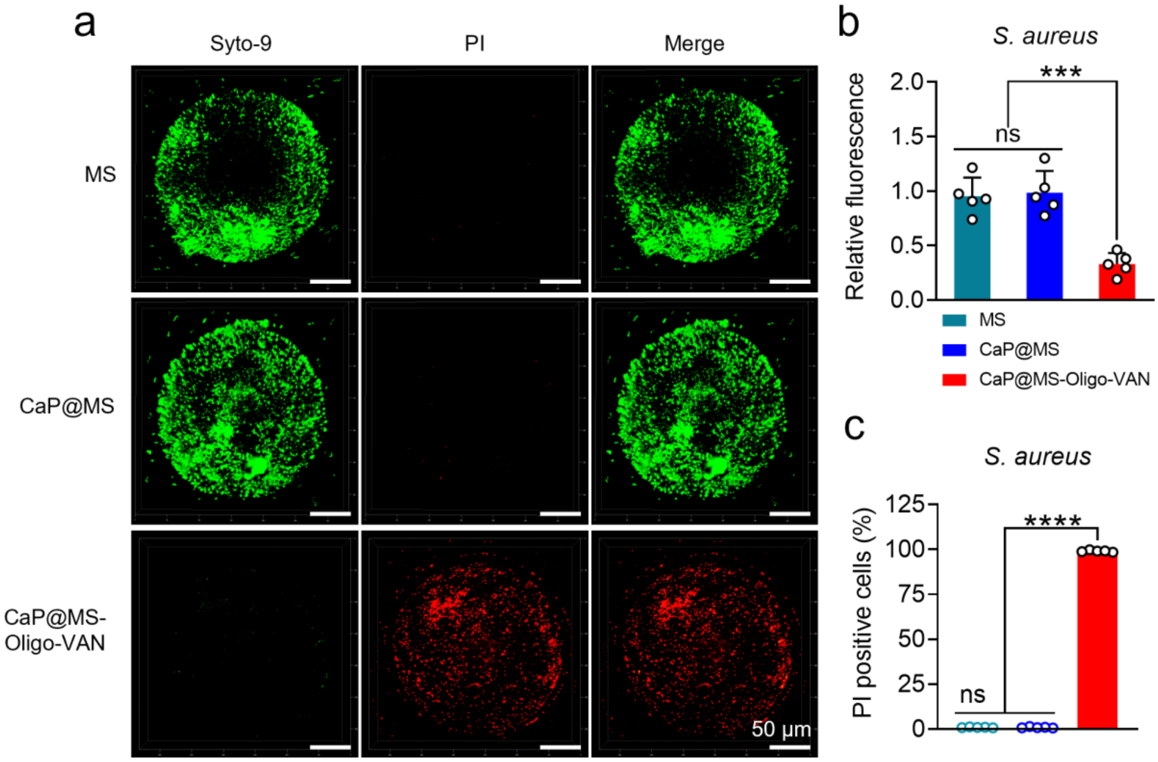

Figure S16. The capture capability of the nano-micro CaP@MS-Oligo-Van composites on *S. aureus* in BHI medium. (a) Representative fluorescence images of live and dead *S. aureus* absorbed on the surface of MS, CaP@MS, and CaP@MS-Oligo-VAN. The microspheres were collected by centrifuging, stained with syto 9/PI, then further observed by CLSM. The visible and dead cells were stained by syto 9 (green) and PI (red), respectively. (b) Quantification of *S. aureus* fluorescence on the surface of MS, CaP@MS, and CaP@MS-Oligo-VAN. (c) Percentage of PI-positive *S. aureus* after MS, CaP@MS, and CaP@MS-Oligo-VAN treatments. Images are representative of five independent experiments. The results in Figure b and c are presented as means ± SD from five independent experiments. *** $P < 0.001$, **** $P < 0.0001$. ns, no significance.

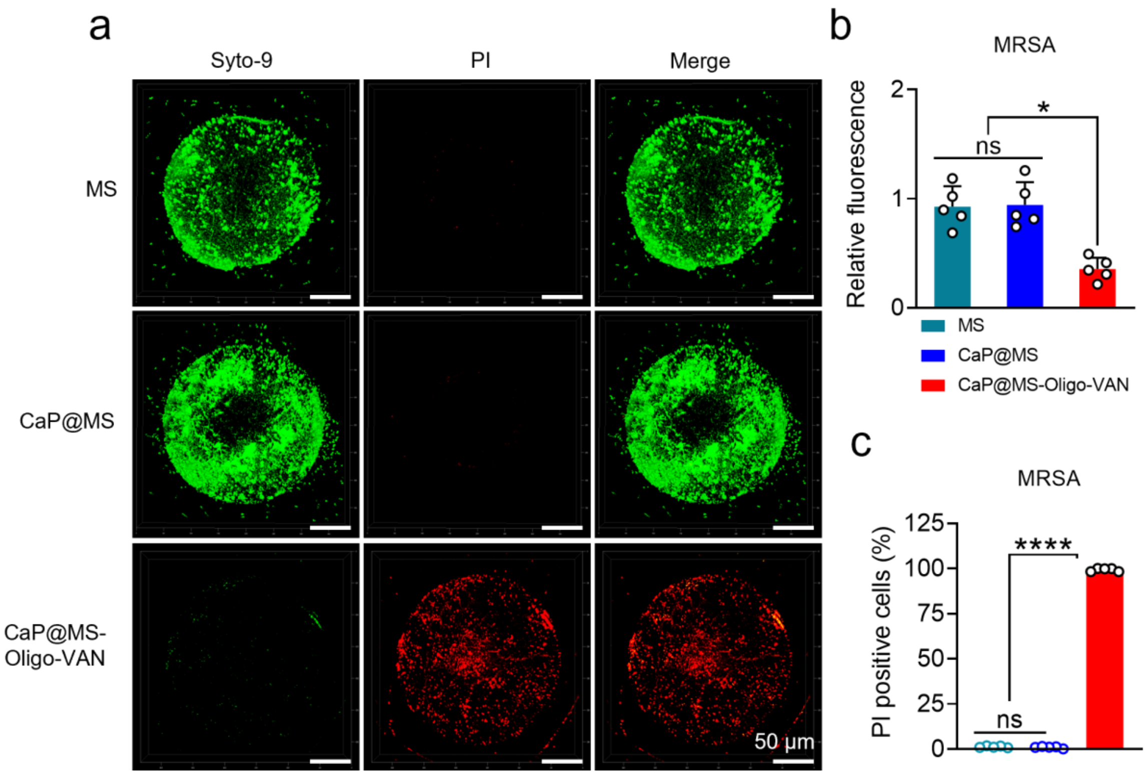

Figure S17. The capture capability of the nano-micro CaP@MS-Oligo-Van composites on MRSA in BHI medium. (a) Representative fluorescence images of live and dead MRSA absorbed on the surface of MS, CaP@MS, and CaP@MS-Oligo-VAN. The microspheres were collected by centrifuging, stained with syto 9/PI, then further observed by CLSM. The visible and dead cells were stained by syto 9 (green) and PI (red), respectively. (b) Quantification of MRSA fluorescence on the surface of MS, CaP@MS, and CaP@MS-Oligo-VAN. (c) Percentage of PI-positive MRSA after MS, CaP@MS, and CaP@MS-Oligo-VAN treatments. Images are representative of five independent experiments. The results in Figure b and c are presented as means ± SD from five independent experiments. * $P < 0.05$, **** $P < 0.0001$. ns, no significance.

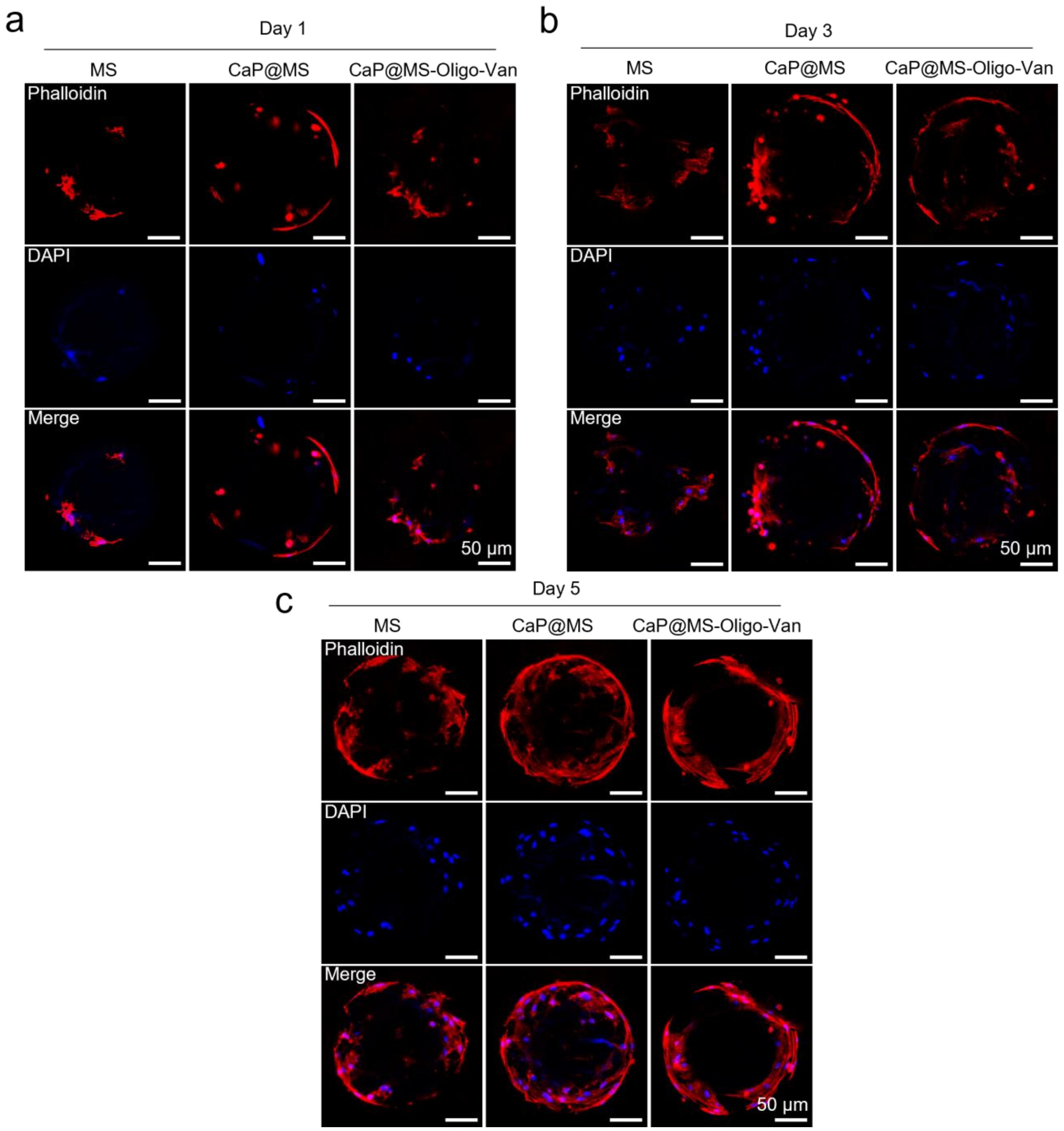

Figure S18. Representative fluorescence images of Phalloidin/DAPI double stained BMSCs treated with MS, CaP@MS, and CaP@MS-Oligo-VAN. The BMSCs were co-incubated with MS, CaP@MS, and CaP@MS-Oligo-VAN for 1 (a), 3 (b), and 5 (c) days, followed by Phalloidin/DAPI staining, and observed by CLSM. Images are representative of five independent experiments.

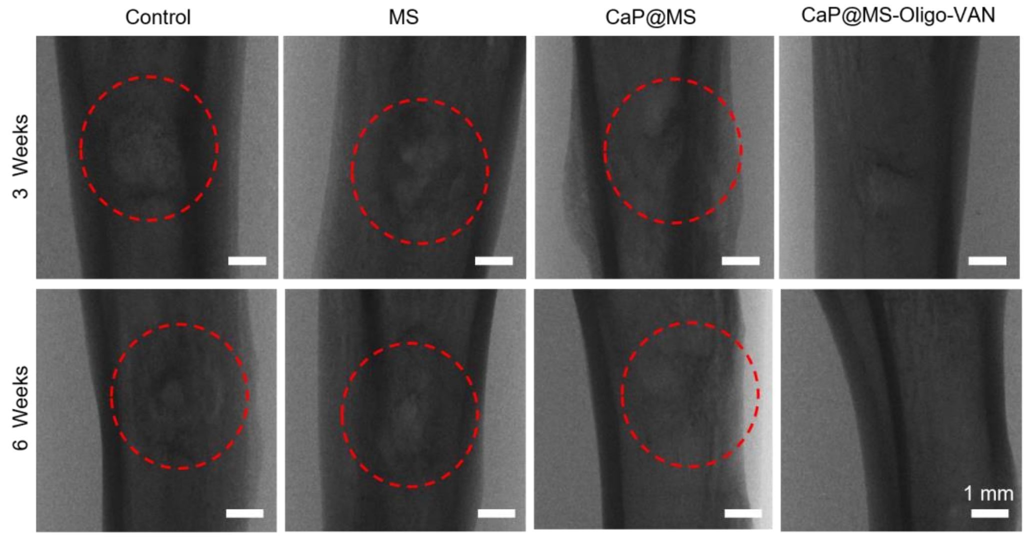

Figure S19. Representative X-Ray images of the infected rat tibias treated with MS, CaP@MS, and CaP@MS-Oligo-VAN for 3 and 6 weeks. Images are representative of five independent experiments.

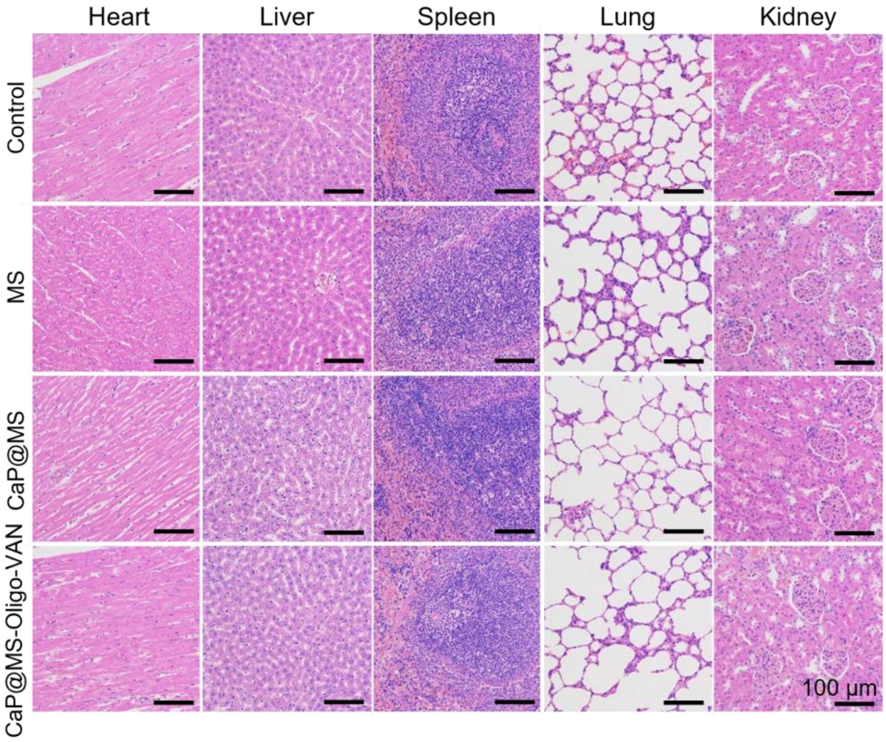

Figure S20. Representative H&E staining images of the major organs (heart, liver, spleen, lung, and kidney) were harvested from normal rats in different groups. The high doses of MS, CaP@MS, and CaP@MS-Oligo-VAN were individually injected into the tibial bone marrow in normal rats. The administration was once a day for 5 continuous days. On day 6, all rats were sacrificed and the organs including hearts, liver, spleens, lungs, and kidneys were excised and processed for H&E staining. Images are representative of five independent experiments.

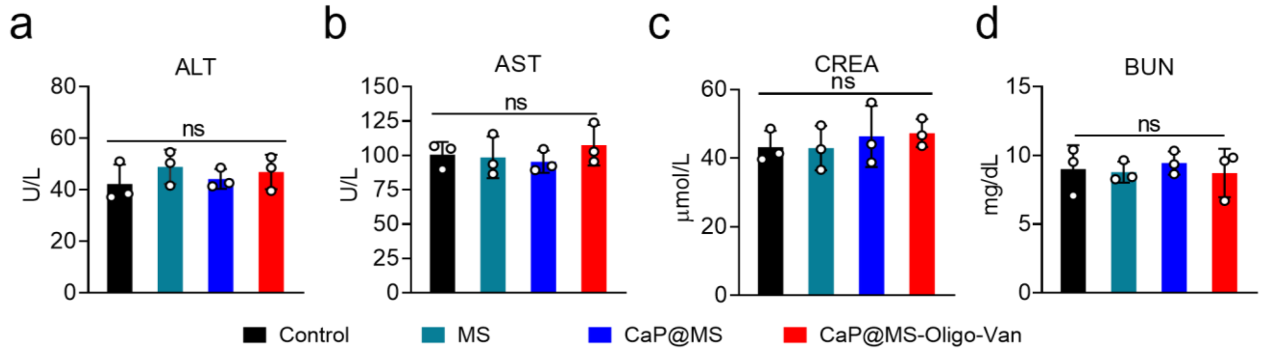

Figure S21. The effects of the nano-micro CaP@MS-Oligo-VAN composites on hepatorenal toxicity in normal rats. The high doses of MS, CaP@MS, and CaP@MS-Oligo-VAN were individually injected into the tibial bone marrow in normal rats. The injection was once a day for 5 continuous days. On day 6, the blood samples were collected into sterile 2-ml heparinized tubes (BD Scientific), and the levels of ALT (a), AST (b), CREA (c), and BUN (d) in serum were determined as indicators of liver and renal function. The results are presented as means ± SD from three independent experiments. ns, no significance.

Table S1. Calculation of vancomycin loading of microspheres

The doses of Van grafted on microspheres (μg/mg) (n=3)		
91.54	93.41	101.03

Table S2. Primers for Real-Time PCR Analysis

Gene		Sequence (5' to 3')
Runx2	Forward	TACCCAGGCGTATTTCAGATGAT
	Reverse	TGTAAGTGAAGGTGGCTGGATAGT
ALP	Forward	TGGTGAGTGACACGGACAAGAA
	Reverse	GCCTGGTAGTTGTTGTGAGCAT
COL-1	Forward	CCCAGCGGTGGTTATGACTT
	Reverse	TCGATCCAGTACTCTCCGCT
OCN	Forward	TGACAAAGCCTTCATGTCCAA
	Reverse	CTCCAAGTCCATTGTTGAGGTAG
